# Squaramide‐Based 5’‐Phosphate Replacements Bind to the DNA Repair Exonuclease SNM1A

**DOI:** 10.1002/slct.201803375

**Published:** 2018-12-04

**Authors:** Eva‐Maria Dürr, William Doherty, Sook Y. Lee, Afaf H. El‐Sagheer, Arun Shivalingam, Peter J. McHugh, Tom Brown, Joanna F. McGouran

**Affiliations:** ^1^ School of Chemistry Trinity Biomedical Sciences Institute Trinity College Dublin 152-160 Pearse St. Dublin 2 Ireland; ^2^ Department of Oncology Weatherall Institute of Molecular Medicine University of Oxford, John Radcliffe Hospital Oxford OX3 9DS UK; ^3^ Department of Chemistry University of Oxford 12 Mansfield Road Oxford OX1 3TA UK; ^4^ Chemistry Branch, Department of Science and Mathematics Faculty of Petroleum and Mining Engineering, Suez University Suez 43721 Egypt

**Keywords:** Exonuclease, metalloenzyme, 5’-modification, oligonucleotide, squaramide

## Abstract

Phosphate groups are often crucial to biological activity and interactions of oligonucleotides, but confer poor membrane permeability. In addition, the group's lability to enzymatic hydrolysis is an obstacle to its use in therapeutics and in biological tools. We present the synthesis of *N*‐oxyamide and squaramide modifications at the 5’‐end of oligonucleotides as phosphate replacements and their biological evaluation using the 5’‐exonuclease SNM1A. The squaryl diamide modification showed minimal recognition as a 5’‐phosphate mimic; however, modest inhibition of SNM1A, postulated to occur through metal coordination at the active site, was observed. Their facile incorporation after solid‐phase synthesis and recognition by the exonuclease makes squaryl diamides attractive neutral 5’‐phosphate replacements for oligonucleotides. This work is the first example of squaryl diamide modifications at the 5’‐terminal position of oligonucleotides and of the potential use of modified oligonucleotides to bind to the metal center of SNM1A.

## Introduction

Modified nucleotides are commonly used as antiviral agents^1^ and oligonucleotides such as siRNA are becoming increasingly popular as potential therapeutics.^2^ Two of the major obstacles in this area are the enzymatic lability of phosphate groups, which serve as key recognition elements, and the poor membrane permeability conferred by them.^3^ Phosphate bioisosteres such as phosphonates, phosphorothioates, boranophosphates and squaramides, are commonly used to overcome these problems and have been the subject of several reviews.^3^, ^4^, ^5^, ^6^ Mononucleotide analogues with isosteres at the 5’‐position are being investigated as antiviral agents, as the introduction of the mimic overcomes the rate limiting phosphorylation of the nucleoside drug.^7^ The activity of siRNA depends on 5’‐terminal phosphorylation by kinases, which limits chemical modifications of siRNA as extensive modification can compromise recognition by kinases.^8^, ^9^, ^10^ The introduction of bioisosteres could circumvent this issue. The introduction of 5’‐(*E*)‐vinylphosphonate in place of a 5’‐phosphate group conferred enhanced activity on single stranded siRNA in cells and animals due to enhanced nuclease resistance.^10^,^11^ Substitution of the phosphate group by 5’‐methylenephosphonate lowered the activity of single stranded siRNA, but conserved it for double stranded siRNA.^8^ A 5’‐malonate group retained or enhanced silencing activity and metabolic stability of siRNA compared to its phosphorylated analogue.^9^ However, the use of terminal 5’‐phosphate replacements on oligonucleotides is challenging, as the mimetics need to withstand solid‐phase oligonucleotide synthesis or be amenable to introduction using mild conditions post solid‐phase synthesis.

In this regard, squaramides are of interest as they are neutral phosphate bioisosteres formed by the reaction of squarate esters with amines. The reaction is highly chemoselective,^12^ facilitating the introduction of amine substituents in aqueous medium and thereby permitting late‐stage diversification. Squaramides have a partial negative charge on the carbonyl oxygen atoms and can coordinate to magnesium ions,^13^ replicating the behavior of a phosphate group. Oligonucleotides containing an internal squaramide form duplexes despite their bent structure at the squaramide linkage.^13^ Mononucleotides with 5’‐squaramides co‐crystallize in the active site of translation initiation factor eIF4E,^14^ and nucleotides containing this group bind to the active site of ADP ribosylhydrolase MDO1.^15^ Squaramides have also been employed as isosteres for phospholipids with potential applications in liposome‐based therapies.^16^ Their widespread use makes them promising candidates as 5’‐phosphate mimetics for short oligonucleotides.

Furthermore, phosphate groups often coordinate to metal ions in proteins, suggesting metal chelators such as hydroxamic acid, used to target metalloproteases,^17^ could also act as phosphate replacements. *N*‐Oxyamides, derivatives of hydroxamic acids, can withstand solid‐phase oligonucleotide synthesis^18^ and potentially bind metals in a similar manner to the phosphate group. Mononucleotides containing amides as phosphate bioisosteres successfully bind to MDO1, supporting this hypothesis.^15^

The objective of this work is the synthesis of oligonucleotides containing 5’‐phosphate replacements, specifically an *N*‐oxyamide and different squaramides, and their evaluation as phosphate isosteres. The DNA repair exonuclease SNM1A, which acts with a 5’‐to‐3’ polarity, was selected since its activity is absolutely dependent upon the presence of a terminal 5’‐phosphate group.^19^ Moreover, SNM1A possesses an unprecedented capacity to digest substrates containing drastically altered bases.^20^,^21^ We have examined whether the replacements act as a substrate for the exonuclease SNM1A or whether they inhibit enzymatic activity.

## Results and Discussion

### Synthesis of modified oligonucleotides

The synthetic targets were designed based on known isosteres and metal‐chelating groups, with the aim of achieving similar molecular recognition by SNM1A through hydrogen bonding or metal coordination compared to the natural 5’‐phosphate group or phosphodiester bond.^22^

**Figure 1:**
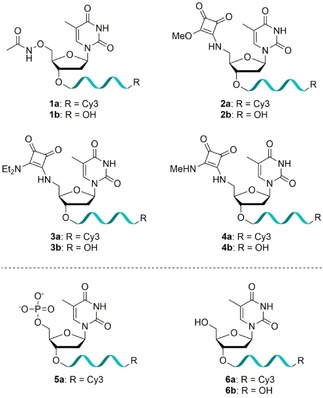
Structure of modified oligonucleotides **1–4** containing phosphate replacements at the 5’‐end, positive control **5 a** with 5’‐phosphate group and negative controls **6 a**‐**b** with 5’‐OH group.

**Scheme 1:**
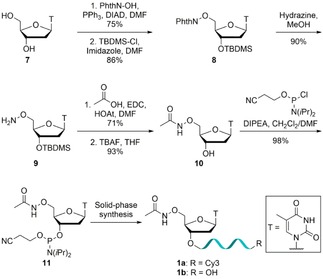
Synthesis of 5’‐oxyamide modified oligonucleotides **1 a**‐**b**.

*N*‐Oxyamide‐containing oligonucleotides **1 a**‐**b**, based on the known metal‐binder hydroxamic acid, and three squaramide derivatives **2 a**‐**4 b**, based on their use as phosphate backbone replacements in DNA,^13^ were synthesized (Figure 1). The introduction of an acetyl protected 5’‐hydroxamic acid group, designed to undergo deprotection after solid‐phase synthesis, was attempted but in our hands no suitable phosphitylating conditions could be identified. The synthesis of the 5’‐oxyamide phosphoramidite **11** was carried out in six steps, giving good to excellent yields (Scheme 1). Oxyamine **9** was prepared from thymidine (**7**) in three steps as reported previously.^23^ Briefly, a Mitsunobu reaction was used to introduce the oxyamine functionality, followed by protecting group manipulations to afford oxyamine **9**. Amide coupling between the oxyamine **9** and acetic acid, followed by TBDMS deprotection, gave alcohol **10**. Subsequent phosphitylation yielded phosphoramidite **11** for incorporation into oligonucleotides. Modified oligonucleotides bearing a 3’‐Cy3 fluorophore **1 a** or a free 3’‐OH group **1 b** were synthesized to allow for visualization where necessary. As the coupling of the modified monomer was not quantitative and separation of the unmodified 20mer from the oxyamide‐containing 21mer proved challenging, the oligonucleotides were used as a 3:2 mixture of 20mer to 21mer **1 a** and a 2:3 mixture of 20mer to 21mer **1 b**. Consequently, observed activity will be underestimated due to the presence of inactive 5’‐OH oligonucleotide.

**Scheme 2:**
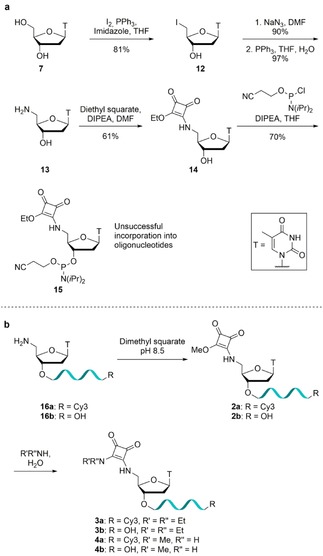
Synthesis of 5’‐modified oligonucleotides. a) Synthesis of 5’‐squaramide modified phosphoramidite **15**. b) Synthesis of squaramide‐modified oligonucleotides **2 a**‐**b**, **3 a**‐**b** and **4 a**‐**b** after solid‐phase synthesis.

The 5’‐squaryl monoamide phosphoramidite **15** was synthesized in five steps, giving moderate to excellent yields, using modified literature procedures (Scheme 2a).^24^, ^25^, ^26^ Intermediate iodothymidine **12** was prepared from thymidine (**7**) using an Appel reaction^24^ before reaction with sodium azide^25^ and Staudinger reduction to give amine **13**. This monomer was reacted with diethyl squarate to give squaryl monoamide **14**,^26^ which was successfully phosphitylated. However, incorporation of phosphoramidite **15** into oligonucleotides in solid‐phase synthesis was unsuccessful. The squaramide modification was therefore introduced by reaction of 5’‐amino oligonucleotides **16 a**‐**b** in aqueous solution with dimethyl squarate to give squaryl monoamide oligonucleotides **2 a**‐**b** with quantitative conversion. Diethylamine or methylamine derivatives were then generated by reaction of **2 a**‐**b** with the desired amine to give squaryl diamides **3 a**‐**b** and **4 a**‐**b** (Scheme 2b). Finally, oligonucleotides containing a 5’‐OH group were synthesized as negative controls (**6 a**‐**b**), with **6 a** phosphorylated enzymatically using T4 polynucleotide kinase to give 5’‐phosphate containing oligonucleotide **5 a** as a positive control for enzymatic activity (Figure 1).

### Evaluation of modified oligonucleotides as substrates for SNM1A

**Figure 2:**
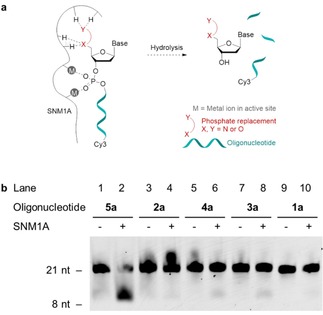
Evaluation of modified oligonucleotides as substrates for SNM1A. a) Modified 3’‐Cy3 labelled oligonucleotides are hydrolyzed if binding to the active site in a similar manner to oligonucleotides containing a 5’‐phosphate group. b) Digestion of oligonucleotides **1 a**‐**5 a** (0.8 pmol, 80 nM) by SNM1A (25 fmol, 2.5 nM) after 60 min. nt=nucleotides.

The Zn^2+^‐dependent exonuclease SNM1A, which has 5’‐to‐3’ activity and requires a terminal 5’‐phosphate for substrate recognition and catalysis,^22^ was used as a model system to evaluate the phosphate mimetic properties of our analogues. The fluorescent modified oligonucleotides **1 a**‐**4 a** and the positive control **5 a** were incubated with SNM1A and their hydrolysis products were analyzed by Urea PAGE. If a substituent in the 5’‐position binds similarly to the natural substrate, hydrolysis of the oligonucleotide backbone is anticipated (i. e. the modified oligonucleotide acts as a substrate) (Figure 2a). 5’‐Phosphorylated oligonucleotide **5 a** is a substrate for SNM1A and is hydrolyzed to eleven nucleotides in size (Figure 2b, lane 2), consistent with Allerston *et* *al*.^22^ No hydrolysis was observed for the squaryl monoamide modified oligonucleotide **2 a** or the *N*‐oxyamide modified oligonucleotide **1 a** (Figure 2b, lanes 4 and 10) while minimal digestion was observed for the squaryl diamide modified oligonucleotides **4 a** and **3 a** relative to the control **5 a** (Figure 2b, lanes 6, 8 and 2). These results suggest that the squaryl diamides were binding in a similar manner to the 5’‐phosphate group present in the natural substrate to a small degree, while oxyamides and squaryl monoamides do not show any recognition in this context. Further attempts to optimize the hydrolysis with regards to concentration of the modified oligonucleotide **4 a** resulted in no observable cleavage when deviating from the conditions shown.

### Evaluation of modified oligonucleotides as inhibitors of SNM1A

**Figure 3:**
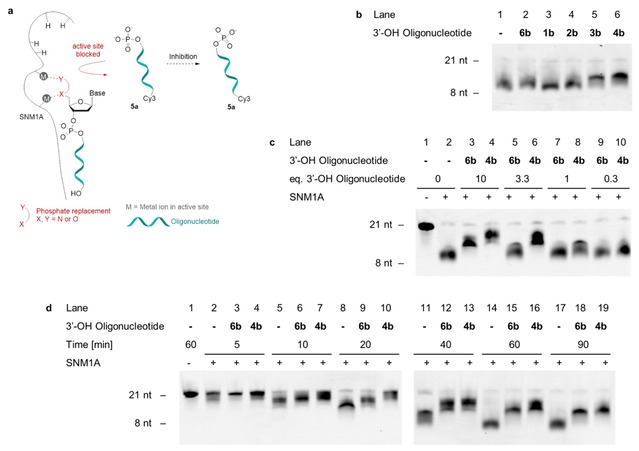
Evaluation of modified oligonucleotides as inhibitors of SNM1A. a) Modified oligonucleotides act as inhibitors if binding to the metal center in the active site, preventing the hydrolysis of 3’‐Cy3‐labelled 5’‐phosphorylated oligonucleotide **5 a**. b) Digestion of oligonucleotide **5 a** (0.8 pmol, 80 nM) by SNM1A (25 fmol, 2.5 nM) in the presence of oligonucleotides **1 b‐4 b** or **6 b** (0.8 pmol, 80 nM) after 60 min. c) Digestion of oligonucleotide **5 a** by SNM1A (25 fmol, 2.5 nM) in the presence of different amounts of oligonucleotide **4 b** or **6 b** relative to **5 a** (0.8 pmol, 80 nM) after 60 min with 5 min preincubation. d) Digestion of oligonucleotide **5 a** by SNM1A (25 fmol, 2.5 nM) in the presence of 3.3 equivalents of oligonucleotide **4 b** or **6 b** (2.7 pmol, 264 nM) relative to **5 a** (0.8 pmol, 80 nM) after varying incubation times with 5 min preincubation. nt = nucleotides.

As an alternative mechanism of action, coordination of the phosphate replacement to the zinc ions in the active site of SNM1A was examined by incubating non‐fluorescent modified oligonucleotides **1 b**‐**4 b** with SNM1A in the presence of fluorescent 5’‐phosphorylated oligonucleotide **5 a**. In cases where the phosphate replacements bind stably to the enzyme active site, they act as inhibitors and hydrolysis of the natural substrate is less efficient (Figure 3a). In this assay, as expected, the 5’‐OH negative control **6 b** did not affect the hydrolysis rate of the fluorescent 5’‐phosphorylated oligonucleotide **5 a** when added in equimolar concentrations compared to the substrate (Figure 3b, lanes 1–2), indicating that any observed inhibitory effect is due to the presence of the 5’‐modification rather than simply due to the presence of additional oligonucleotides. One equivalent of modified oligonucleotide compared to the substrate led to decreased enzyme activity for the squaryl diamides **3 b** and **4 b** (Figure 3b, lanes 5–6), but not for oxyamide **1 b** and squaryl monoamide **2 b** (Figure 3b, lanes 3–4). This suggests that squaryl diamides **3 b** and **4 b** form stable complexes with the metal center, blocking the enzyme active site, analogous to the binding of the cephalosporin ceftriaxone.^27^ This inhibitor was shown to bind to the metal center in SNM1A through two carbonyl groups when co‐crystallized with SNM1A.^28^ Next, a concentration‐dependence study was carried out with oligonucleotide **4 b** (Figure 3c) to explore this finding. Firstly, for the negative control **6 b**, a decrease in the hydrolysis rate was observed when present in concentrations above one equivalent compared to the phosphorylated oligonucleotide (Figure 3c, lanes 3 and 5). However, this effect was less than with the modified oligonucleotide **4 b** (Figure 3c, lanes 4 and 6). The most pronounced difference after 60 min was observed at 3.3 equivalents of modified oligonucleotide (Figure 3c, lanes 4 and 6). Inhibition is not observed when the stoichiometry is below one equivalent (Figure 3c, lane 10).

Analysis of the reaction progress over time shows that the reaction is near completion after 60 minutes (Figure 3d, lane 14). There is a notable difference in the rate of hydrolysis of 5’‐phosphorylated oligonucleotide **5 a** between the reaction without additional oligonucleotide, with the 5’‐OH negative control **6 b** and with the modified oligonucleotide **4 b**. The addition of either oligonucleotide leads to a decrease in hydrolysis rate, visible from 10 minutes onwards (Figure 3d, lanes 5–19). However, the modified oligonucleotide **4 b** has a greater impact on the rate than the 5’‐OH negative control **6 b** between 20 and 60 minutes incubation (Figure 3d, lanes 8–16), with the biggest contrast after 60 minutes (Figure 3d, lanes 14–16). The same effects were observed for squaryl diamide **3 b** (Figures S1‐S2 in Supporting Information), suggesting that there is no significant difference between the binding of the secondary squaryl diamide **4 b** and the tertiary squaryl diamide **3 b**.

**Figure 4:**
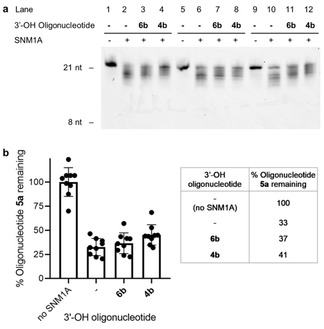
Evaluation of modified oligonucleotide **4 b** as an inhibitor of SNM1A. a) Representative digestion of oligonucleotide **5 a** by SNM1A (25 fmol, 2.5 nM) in the presence of 3.3 equivalents of oligonucleotide **4 b** or **6 b** (2.7 pmol, 264 nM) relative to **5 a** (0.8 pmol, 80 nM) after 10 min with 5 min preincubation. b) Quantification of digestion of oligonucleotide **5 a** by SNM1A (25 fmol, 2.5 nM) in the presence of 3.3 equivalents of oligonucleotide **4 b** or **6 b** (2.6 pmol, 264 nM) relative to **5 a** (0.8 pmol, 80 nM) after 10 min with 5 min preincubation. Values represent the mean ±SD carried out for *n*=9 independent experiments performed in triplicate. nt=nucleotides.

For quantification of the effect of the squaramide‐modification, the hydrolysis of 5’‐phosphorylated oligonucleotide **5 a** in the presence and absence of 5’‐OH or 5’‐modified oligonucleotides **6 b** and **4 b** after 10 minutes was analyzed by Urea PAGE (Figure 4a). At this time point the reaction is still in the linear range. The efficiency of hydrolysis was determined by measuring the amount of fluorescent substrate **5 a** remaining after 10 minutes incubation with SNM1A relative to a control reaction without SNM1A. Nine independent experiments were carried out in triplicate (Figure 4a and S3). Our results show that in the absence of any additional oligonucleotide, 33% of substrate **5 a** remained after the reaction. While the addition of 5’‐OH oligonucleotide **6 b** increased this to 37%, the addition of 5’‐modified oligonucleotide **4 b** showed a slightly stronger effect, with 41% of oligonucleotide **5 a** present after the reaction (Figure 4b).

The difference in inhibition between the squaramide‐modified oligonucleotide **4 b** and 5’‐OH oligonucleotide **6 b** illustrates a modest inhibitory effect of the squaramide moiety. This is postulated to occur through the squaramide binding to the metal center of SNM1A. The observed inhibition and proposed binding is consistent with the previously reported metal binding of squaramides^13^ and the binding mode of ceftriaxone to the metal center in SNM1A through two carbonyl groups.^28^

## Conclusions

In conclusion, we report the synthesis of four different 5’‐modifications of deoxyoligonucleotides. An *N*‐oxyamide modification was introduced at the 5’‐position of a nucleoside and incorporated using solid‐phase oligonucleotide synthesis, while three squaramide modifications were introduced chemoselectively after solid‐phase synthesis to circumvent difficulties arising during solid‐phase oligonucleotide synthesis. This strategy permits access to a wide range of substituted squaramides that can be introduced post‐solid‐phase, allowing for late stage diversification and circumventing the requirement for compatibility with solid‐phase synthesis. The recognition and binding mode of the phosphate replacements was examined using the exonuclease SNM1A, showing minimal recognition through hydrogen bonding for both squaryl diamide modifications. Inhibition was observed for the same squaryl diamides, postulated to be enhanced through metal coordination of the squaramide moiety. Our results demonstrate a novel synthetic methodology for the synthesis of 5’‐squaramide modified oligonucleotides and illustrates their potential use as metal‐binding phosphate substitutes. The facile introduction of the group as well as of different substituents makes squaramides attractive 5’‐modifications for the wider scientific community.

### Supporting Information Summary

The supporting information contains procedures for the synthesis of modified nucleosides, NMR spectra, procedures for oligonucleotide synthesis, mass spectra of oligonucleotides, procedures for enzymatic assays and additional figures.

## Conflict of interest

The authors declare no conflict of interest.

## Supporting information

As a service to our authors and readers, this journal provides supporting information supplied by the authors. Such materials are peer reviewed and may be re‐organized for online delivery, but are not copy‐edited or typeset. Technical support issues arising from supporting information (other than missing files) should be addressed to the authors.

Supplementary
