# Supplementary material for: Squaramide‐Based 5’‐Phosphate Replacements Bind to the DNA Repair Exonuclease SNM1A
Source: ChemistrySelect. 2018 Dec 4;3(45):12824–9. doi: 10.1002/slct.201803375 (PMC6685075; doi:10.1002/slct.201803375)
Supplement: Supplementary file 1 — Supplementary [file SLCT-3-12824-s001.pdf]

Chemistry SELECT

Supporting Information

© Copyright Wiley-VCH Verlag GmbH & Co. KGaA, 69451 Weinheim, 2019

Squaramide-Based 5'-Phosphate Replacements Bind to the DNA Repair Exonuclease SNM1A

Eva-Maria Dürr, William Doherty, Sook Y. Lee, Afaf H. El-Sagheer, Arun Shivalingam, Peter J. McHugh, Tom Brown, and Joanna F. McGouran*

Supporting Information

1. **Synthesis of modified nucleosides S2**
2. **NMR spectra S10**
3. **Oligonucleotide synthesis S17**
4. **Mass spectra of oligonucleotides S18**
5. **Biological evaluation – procedures and supplementary figures S24**
6. **References S26**

1. Synthesis of modified nucleosides

General experimental methods

^{1}H, ^{13}C and ^{31}P NMR spectra were recorded on Bruker 400 MHz or 600 MHz system spectrometers in DMSO-d_6 or $CDCl_3$ relative to residual DMSO (δ = 2.50 ppm) or $CHCl_3$ (δ = 7.26 ppm). Chemical shifts are reported in ppm and coupling constants are reported in Hertz (Hz) accurate to 0.2 Hz. NMR spectra were assigned using HSQC, HMBC, DEPT and EXSY experiments. Modified nucleosides are numbered according to standard nucleoside convention. Mass spectrometry measurements were carried out on a Bruker ESI or APCI HRMS. Melting points were measured using a Griffin melting point apparatus and are uncorrected. Infrared (IR) spectra were obtained on a Perkin Elmer spectrophotometer. Flash column chromatography was carried out using silica gel, particle size 0.04-0.063 mm. TLC analysis was performed on precoated $60F_{254}$ slides and visualised by UV irradiation, potassium permanganate stain (3 g $KMnO_4$, 20 g K_2CO_3, 300 mL H_2O), ninhydrin stain (1.5 g ninhydrin, 5 mL AcOH, 500 mL 95% EtOH), anisaldehyde stain (9.2 mL *p*-methoxybenzaldehyde, 3.75 mL AcOH, 338 mL 95% EtOH, 12.5 mL conc. H_2SO_4) and iodine. THF and CH_2Cl_2 were dried using a PureSolv MD solvent purification system. Petroleum ether refers to the fraction of petroleum ether that boils at 40–60 °C. Chemicals were purchased from Acros Organics, Aldrich, Alfa Aesar Carbosynth, Fisher Scientific, Fluorochem and Sigma Aldrich and used as purchased without further purification.

5'-*O*-Phthalimidothymidine (16)

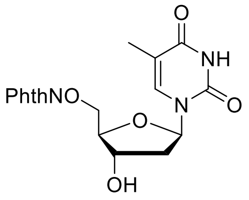

16

Thymidine **7** (5.09 g, 21.0 mmol) was dissolved in dry DMF (55 mL) under argon and cooled to 0 °C. PPh_3 (7.04 g, 26.8 mmol) and *N*-hydroxyphthalimide (4.49 g, 27.5 mmol) were added followed by the dropwise addition of DIAD (6.1 mL, 31.1 mmol) in DMF (10 mL). The reaction mixture was warmed to r.t. and stirred for 18 h. After this time, TLC analysis (CH_2Cl_2-MeOH, 9:1) showed the complete consumption of the starting material (R_f = 0.3) and the formation of the product (R_f = 0.7). The solution was concentrated and the resulting oil dissolved in CH_2Cl_2 (50 mL). The solution was poured into a mixture of H_2O-ice (250 mL) and stirred for 30 minutes. The resulting precipitate was collected by vacuum filtration and washed with cold EtOH (3 x 50 mL) to afford the desired product **16** as a white powder (6.11 g, 75%).

ν_{max}/cm^{-1} (neat) 3449 (O-H), 3234 (N-H), 3082 (C-$H_{ar)}$, 2929 (C-H), 1786 (C=O), 1728 (C=O), 1680 (C=O), 1648 (C=C).
^{1}H NMR (400 MHz, DMSO-d_6): δ = 1.80 (s, 3 H, CH_3^T), 2.10 - 2.14 (m, 2 H, H-2'a, H-2'b), 4.07-4.10 (m, 1 H, H-4'), 4.36 (d, $J_{4',5'}$ = 4.4 Hz, 2 H, H-5'a, H-5'b), 4.39 (m, 1 H, H-3'), 5.46 (d, $J_{3',OH}$ = 3.8 Hz, 1 H, OH), 6.19-6.23 (m, 1 H, H-1'), 7.58 (s, 1 H, H-6), 7.84 - 7.87 (m, 4 H, Phth), 11.29 (s, 1 H, NH) ppm.
^{13}C {^{1}H} NMR (100 MHz, DMSO-d_6) δ = 12.1 (CH_3^T), 38.8 (C-2'), 70.7 (C-3'), 77.7 (C-5'), 84.1 (C-4'), 84.3 (C-1'), 109.9 (C-5), 123.3 (Phth), 128.6 (Phth), 134.8 (Phth), 135.8 (C-6), 150.4 (C-2), 163.0 (CO Phth), 163.7 (C-4) ppm.
HRMS (ESI^+): *m/z* calc. 410.0959 $[M + Na]^+$, found: 410.0958
The spectroscopic data are in agreement with those reported in the literature.[1]

3′-*O*-(*tert*-Butyldimethylsilyl)-5′-*O*-phthalimidothymidine (8)

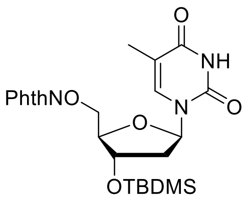

8

5′-*O*-Phthalimidothymidine **16** (2.96 g, 7.7 mmol) was dissolved in dry DMF (15 mL) under argon and imidazole (1.37 g, 20.1 mmol) was added. A solution of TBDMS-Cl (1.52 g, 10.1 mmol) in dry DMF (15 mL) was added dropwise and the reaction mixture was stirred at r.t. for 18 h. After this time, TLC analysis (EtOAc) showed the consumption of the starting material (R_f = 0.3) and the formation of the product (R_f = 0.8). The solution was diluted with brine (150 mL) and extracted with EtOAc (200 mL). The organic layer was washed with brine (2 x 50 mL), dried over Na_2SO_4, filtered and concentrated. Purification by flash column chromatography (petroleum ether-EtOAc, 1:1) afforded the desired product **8** as a white powder (3.31 g, 86%).

ν_{max}/cm^{-1} (neat) 2930 (C-H), 2856 (C-H), 1790 (C=O), 1701 (C=O), 1665 (C=C).
^{1}H NMR (400 MHz, DMSO-d$_6$): δ = 0.12 (s, 6 H, 2 x CH_3^{TBDMS}), 0.88 (s, 9 H, *t*-Bu^{TBDMS}), 1.80 (d, $J_{CH3T,6}$ = 0.9 Hz, 3 H, CH_3^T), 2.09 (ddd, $J_{2'a,3'}$ = 2.6 Hz, $J_{1',2'a}$ = 6.1 Hz, $J_{2'a,2'b}$ = 13.6 Hz, 1 H, H-2′a), 2.25 (ddd, $J_{2'b,3'}$ = 6.0 Hz, $J_{1',2'b}$ = 8.0 Hz, $J_{2'a,2'b}$ = 13.6 Hz, 1 H, H-2′b), 4.07-4.10 (m, 1 H, H-4′), 4.35-4.37 (m, 2 H, H-5′a, H-5′b), 4.60-4.63 (m, 1 H, H-3′), 6.19 (dd, $J_{1',2'a}$ = 6.1 Hz, $J_{1',2'b}$ = 8.0 Hz, 1 H, H-1′), 7.56 (d, $J_{CH3T,6}$ = 0.9 Hz, 1 H, H-6), 7.84 - 7.89 (m, 4 H, Phth), 11.30 (s, 1 H, NH) ppm.
^{13}C {^{1}H} NMR (100 MHz, DMSO-d$_6$): δ = -4.88 (CH_3^{TBDMS}), -4.94 (CH_3^{TBDMS}), 12.1 (CH_3^T), 17.7 (qC, *t*-Bu^{TBDMS}), 25.7 (*t*-Bu^{TBDMS}), 39.0 (C-2′), 72.3 (C-3′), 77.1 (C-5′), 84.1 (C-4′), 84.3 (C-1′), 109.8 (C-5), 123.3 (Phth), 128.5 (qC, Phth), 134.8 (Phth), 135.8 (C-6), 150.4 (C-2), 162.9 (CO Phth), 163.6 (C-4) ppm.
HRMS (ESI$^+$): *m/z* calc. 524.1823 [M + Na]$^+$, found: 524.1825
The spectroscopic data are in agreement with those reported in the literature. [1]

5′-*O*-Amino-3′-*O*-(*tert*-butyldimethylsilyl)thymidine (9)

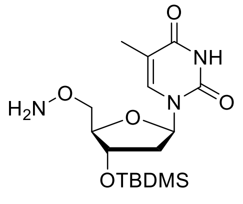

9

Protected 5′-*O*-Phthalimidothymidine **8** (2.00 g, 4.0 mmol) was suspended in MeOH (16 mL) and hydrazine hydrate solution (750 µl, 80%, 12.3 mmol) was added. The reaction mixture became clear, followed by the formation of a precipitate. After 3.5 h stirring at r.t., TLC analysis (petroleum ether-EtOAc, 1:1) showed the complete consumption of the starting material (R_f = 0.4) and the formation of the product (R_f = 0.1). The suspension was diluted with Et_2O (80 mL) and washed with sat. aq. $NaHCO_3$ (80 mL). The aqueous layer was extracted with Et_2O (3 x 150 mL), dried over Na_2SO_4, filtered and concentrated to afford the desired product **9** as a white solid (1.37 g, 90%). The crude product was carried forward without further purification.

ν_{max}/cm^{-1} (neat) 3222 (N-H), 2930 (C-H), 2858 (C-H), 1662 (C=O).
^{1}H NMR (400 MHz, $CDCl_3$): δ = 0.08 (s, 6 H, 2 x CH_3^{TBDMS}), 0.89 (s, 9 H, *t*-Bu^{TBDMS}) 1.93 (d, $J_{CH3T,6}$ = 1.0 Hz, 3 H, CH_3^{T}), 2.08 (app. dt, *J* = 6.5 Hz, *J* = 13.4 Hz, 1 H, H-2'a), 2.26 (ddd, $J_{2'b,3'}$ = 3.7 Hz, $J_{1',2'b}$ = 6.5 Hz, $J_{2'a,2'b}$ = 13.4 Hz, 1 H, H-2'b), 3.85 (dd, $J_{4',5'a}$ = 4.4 Hz, $J_{5'a,5'b}$ = 11.0 Hz, 1 H, H-5'a), 3.96 (dd, $J_{4',5'b}$ = 3.1 Hz, $J_{5'a,5'b}$ = 11.0 Hz, 1 H, H-5'b), 4.00-4.03 (m, 1 H, H-4'), 4.37 (app. dt, *J* = 3.7 Hz, *J* = 6.5 Hz, 1 H, H-3'), 6.24 (app. t, *J* = 6.5 Hz, 1 H, H-1'), 7.38 (d, 1 H, $J_{CH3T,6}$ = 1.0 Hz, H-6), 8.53 (bs, 1 H, NH) ppm.
^{13}C {^{1}H} NMR (100 MHz, $CDCl_3$): δ = -4.7 (CH_3^{TBDMS}), -4.6 (CH_3^{TBDMS}), 12.9 (CH_3^{T}), 18.1 (qC, *t*-Bu^{TBDMS}), 25.8 (*t*-Bu^{TBDMS}), 41.1 (C-2'), 72.0 (C-3'), 75.5 (C-5'), 85.5 (C-1'), 85.6 (C-4'), 111.0 (C-5), 135.9 (C-6), 150.3 (C-2), 163.7 (C-4) ppm.
HRMS (ESI^+): *m/z* calc. 372.1949 $[M + H]^+$, found: 372.1940
The spectroscopic data are in agreement with those reported in the literature. [1]

N-acetyl-5'-O-amino-3'-O-(tert-butyldimethylsilyl)thymidine (17)

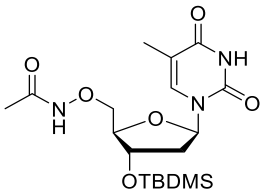

17

AcOH (57 µl, 1.00 mmol) was dissolved in dry DMF (10 mL) under argon and cooled to 0 °C. HOAt (166 mg, 1.22 mmol) and EDC (213 mg, 1.11 mmol) were added and the reaction mixture was stirred for 10 minutes. Amine **9** (350 mg, 0.94 mmol) was added and the reaction mixture was warmed to r.t. and stirred for 24 h. After this time, TLC analysis (CH_2Cl_2-MeOH, 19:1) showed the consumption of the starting material (R_f = 0.4) and the formation of the product (R_f = 0.3). The reaction mixture was diluted with EtOAc (100 mL) and washed with sat. aq. $NaHCO_3$ (100 mL). The aqueous layer was extracted with EtOAc (2 x 100 mL) and the combined organic extracts were washed with brine (100 mL), dried over Na_2SO_4, filtered and concentrated. Flash column chromatography (CH_2Cl_2-MeOH, 19:1) afforded the desired product **17** as a white solid (277 mg, 71%).

ν_{max}/cm^{-1} ($CDCl_3$) 3222 (N-H), 2930 (C-H), 2878 (C-H), 1662 (C=O).
^{1}H NMR (400 MHz, $CDCl_3$): δ = 0.09 (s, 6 H, 2 x CH_3^{TBDMS}), 0.89 (s, 9 H, *t*-Bu^{TBDMS}), 1.92 (s, 3 H, CH_3^{Ac}), 1.93 (s, 3 H, CH_3^{T}), 2.20-2.30 (m, 2 H, H-2'a, H-2'b), 4.01 (dd, $J_{3',4'}$ = 2.7 Hz, $J_{4',5'a}$ = 3.5 Hz, 1 H, H-4'), 4.07 (dd, $J_{4',5'a}$ = 3.5 Hz, $J_{5'a,5'b}$ = 10.9 Hz, 1 H, H-5'a), 4.16 (d, $J_{5'a,5'b}$ = 10.9 Hz, 1 H, H-5'b), 4.59 (m, 1 H, H-3'), 6.15 (app. t, *J* = 6.8 Hz, 1 H, H-1'), 7.45 (s, 1 H, H-6), 8.58-8.72 (m, 2 H, NH^{T}, CO-NH-O) ppm.
^{13}C {^{1}H} NMR (100 MHz, $CDCl_3$): δ = -4.8 (CH_3^{TBDMS}), -4.6 (CH_3^{TBDMS}), 12.5 (CH_3^{T}), 18.0 (qC, *t*-Bu^{TBDMS}), 19.9 (CH_3^{Ac}), 25.8 (*t*-Bu^{TBDMS}), 40.5 (C-2'), 71.9 (C-3'), 75.3 (C-5'), 85.6 (C-4'), 86.5 (C-1'), 111.3 (C-5), 137.1 (C-6), 150.7 (C-2), 164.5 (C-4), 168.0 (CO-NH-O) ppm.
HRMS (ESI^+): *m/z* calc. 436.1874 $[M + Na]^+$, found: 436.1864

***N*-acetyl-5'-*O*-aminothymidine (10)**

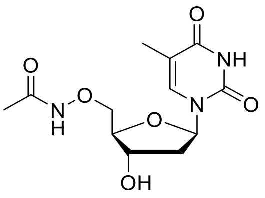

10

Protected nucleoside **17** (200 mg, 48 µmol) was dissolved in THF (5.0 mL). Tetra-*n*-butylammonium fluoride trihydrate (275 mg, 87 µmol) was added and the solution was stirred at r.t. for 17 h. After this time, TLC analysis (CH_2Cl_2-MeOH, 9:1) showed the complete consumption of the starting material (R_f = 0.4) and the formation of the product (R_f = 0.2). The suspension was concentrated and purification by flash column chromatography (CH_2Cl_2-MeOH, 9:1) afforded the desired product **10** as a white solid (104 mg, 72%).

ν_{max}/cm^{-1} (neat) 3263 (O-H), 3191 (N-H), 3022(C-H_{ar}), 1647 (C=O).
^{1}H NMR (600 MHz, DMSO-d_6): δ = 1.73 (s, 3 H, CH_3^{Ac}), 1.80 (s, 3 H, CH_3^{T}), 2.06-2.08 (m, 2 H, H-2'a, H-2'b), 3.89 (dd, $J_{4',5'a}$ = 5.4 Hz, $J_{5'a,5'b}$ = 10.0 Hz, 1 H, H-5'a), 3.92-3.94 (m, 1 H, H-4'), 3.98 (dd, $J_{4',5'b}$ = 2.9 Hz, $J_{5'a,5'b}$ = 10.0 Hz, 1 H, H-5'b), 4.26-4.28 (m, 1 H, H-3'), 5.37 (bs, 1 H, OH), 6.19 (app. t, *J* = 7.0 Hz, 1 H, H-1'), 7.65 (s, 1 H, H-6), 11.23 (m, 2 H, NH^T, CO-NH-O) ppm.
^{13}C {^{1}H} NMR (151 MHz, DMSO-d_6): δ = 12.1 (CH_3^{T}), 19.6 (CH_3^{Ac}), 39.1 (C-2'), 70.7 (C-3'), 75.7 (C-5'), 84.0 (C-4'), 84.1 (C-1'), 109.8 (C-5), 136.1 (C-6), 150.5 (C-2), 163.8 (C-4), 166.6 (CO-NH-O) ppm.
HRMS (ESI^+): *m/z* calc. 322.1010 $[M + Na]^+$, found: 322.1011

***N*-acetyl-5'-O-aminothymidine-3'-2-cyanoethyl diisopropylphosphoramidite (11)**

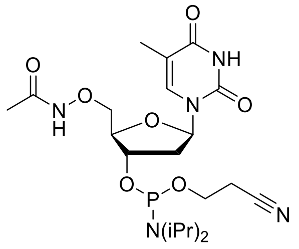

11

Alcohol **10** (71 mg, 273 µmol) was coevaporated with dry pyridine (3 x 4.0 mL), dried under high vacuum and dissolved in a mixture of dry CH_2Cl_2 (3.0 mL) and dry DMF (0.5 mL) under argon. Molecular sieves (3 Å) were added followed by the addition of DIPEA (100 µL, 574 µmol). The flask was purged with argon and 2-Cyanoethyl *N,N*-diisopropylchlorophosphoramidite (110 µL, 285 µmol) was added slowly in a dropwise manner and the reaction mixture was purged with argon. After stirring at r. t. for 2.5 h, TLC analysis (CH_2Cl_2-MeOH-pyridine, 90:10:0.5) showed the presence of starting material (R_f = 0.2). A further portion of DIPEA (70 µL, 402 µmol) was added followed by a further portion of 2-Cyanoethyl *N,N*-diisopropylchlorophosphoramidite (60 µL, 269 µmol). After a further 1.5 h, TLC analysis still showed the presence of starting material and a further portion of DIPEA (70 µL, 402 µmol) was added followed by a further portion of 2-Cyanoethyl *N,N*-diisopropylchlorophosphoramidite (60 µL, 269 µmol). After stirring at r.t. for 1 h, TLC analysis still showed the presence of starting material and a further portion of DIPEA (70 µL, 402 µmol) was added followed by a further portion of 2-Cyanoethyl *N,N*-diisopropylchlorophosphoramidite (60 µL, 269 µmol). After stirring at r.t. for 1 h and a total reaction time of 6 h, TLC analysis showed the complete consumption of the starting material and the formation of the product. The reaction

mixture was diluted with degassed EtOAc (30 mL) and washed with degassed sat. aq. KCl. The aqueous layer was extracted with degassed EtOAc (20 mL) and the combined organic layers were dried over Na_2SO_4 and concentrated. Purification by flash column chromatography (EtOAc-pyridine 99.5:0.5) afforded the desired product **11** as an oil (118 mg, 98%). The formation of the phosphoramidite was confirmed by ^{31}P NMR and the phosphoramidite was used in solid-phase synthesis without further characterisation.

^{31}P NMR (162 MHz, DMSO-d_6): δ = 147.6, 147.8 ppm.

5′-Deoxy-5′-iodothymidine (12)

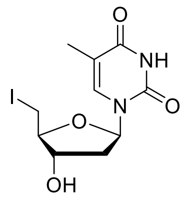

12

Thymidine **7** (20.0 g, 82.6 mmol) was suspended in dry THF (300 mL) under argon. PPh_3 (26.0 g, 99.1 mmol) and imidazole (7.0 g, 102.8 mmol) were added and the reaction mixture was cooled to 0 °C. A solution of iodine (23.0 g, 90.6 mmol) in dry THF (100 mL) was added dropwise and the reaction mixture was slowly warmed to r.t. and stirred for 18 h. After this time, TLC analysis (CH_2Cl_2-MeOH, 9:1) showed the complete consumption of the starting material (R_f = 0.2) and the formation of the product (R_f = 0.4). The reaction was quenched by the addition of H_2O (100 mL). THF was removed *in vacuo* and EtOAc (300 mL) was added. The desired product **12** was collected as a white precipitate by vacuum filtration. The layers of the filtrate were separated and the aqueous layer was extracted with EtOAc (4 x 100 mL). The combined organic extracts were concentrated to approx. 100 mL to afford further product as a white precipitate which was collected by vacuum filtration. The combined precipitates were recrystallized from EtOH to give the desired product **12** as a white crystalline solid (23.0 g, 81%); mp 166-168 °C dec. (EtOH) (lit.[2] 170-173 °C).

ν_{max}/cm^{-1} (neat) 3462 (O-H), 3145 (N-H), 3018 (C-H_{ar}), 2816 (C-H), 1698 (C=O), 1665 (C=O).
^1H NMR (600 MHz, DMSO-d_6): δ = 1.80 (s, 3 H, CH_3^T), 2.08 (ddd, $J_{2'a,3'}$ = 3.1 Hz, $J_{1',2'a}$ = 6.4 Hz, $J_{2'a,2'b}$ = 13.5 Hz, 1 H, H-2'a), 2.29 (ddd, $J_{2'b,3'}$ = 6.4 Hz, $J_{1',2'b}$ = 7.8 Hz, $J_{2'a,2'b}$ = 13.5 Hz, 1 H, H-2'b), 3.39 (dd, $J_{4',5'a}$ = 6.2 Hz, $J_{5'a,5'b}$ = 10.4 Hz, 1 H, H-5'a), 3.52 (dd, $J_{4',5'b}$ = 6.2 Hz, $J_{5'a,5'b}$ = 10.4 Hz, 1 H, H-5'b), 3.81 (app. td, *J* = 3.0 Hz, *J* = 6.2 Hz, 1 H, H-4'), 4.19 (app. dt, *J* = 3.0 Hz, *J* = 6.4 Hz, 1 H, H-3'), 5.49 (bs, 1 H, OH), 6.22 (dd, $J_{1',2'a}$ = 6.4 Hz, $J_{1',2'b}$ = 7.8 Hz, 1 H, H-1'), 7.52 (s, 1 H, H-6), 11.33 (s, 1 H, NH) ppm.
^{13}C {^1H} NMR (151 MHz, DMSO-d_6): δ = 7.9 (C-5'), 12.2 (CH_3^T), 37.9 (C-2'), 73.0 (C-3'), 84.0 (C-1'), 85.4 (C-4'), 109.9 (C-5), 136.2 (C-6), 150.5 (C-2), 163.7 (C-4) ppm.
HRMS (APCI$^+$): *m/z* calc. 352.9998 $[M + H]^+$, found: 352.9992
The spectroscopic data are in agreement with those reported in the literature.[3]

5′-Azido-5′-deoxythymidine (18)

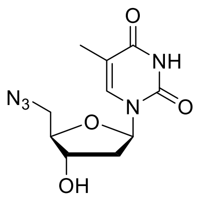

18

5′-Deoxy-5′-iodothymidine **12** (3.52 g, 10.0 mmol) was dissolved in dry DMF (50 mL) under argon. After NaN_3 (2.04 g, 31.4 mmol) was added, the reaction mixture was heated to 60 °C and stirred for 17 h. After this time, TLC analysis (CH_2Cl_2-MeOH, 9:1) showed the complete consumption of the starting material ($R_f = 0.6$) and the formation of the product ($R_f = 0.5$). The reaction mixture was cooled to r.t., diluted with H_2O (30 mL) and extracted with EtOAc (12 x 50 mL). The combined organic layers were washed with brine (50 mL), dried over $MgSO_4$, filtered and concentrated. The residue was recrystallised from MeOH to afford the desired product **18** as a white crystalline solid (2.39 g, 90%); mp 156-157 °C (MeOH) (lit.[4] 157-159 °C).

ν_{max}/cm^{-1} (neat) 3382 (O-H), 3184 (N-H), 3049 (C-H_{ar}), 2928 (C-H), 2094 (N=N=N), 1716 (C=O), 1649 (C=O).
^1H NMR (600 MHz, DMSO-d_6): δ = 1.79 (d, $J_{CH3T,6}$ = 1.2 Hz, 3 H, CH_3^T), 2.08 (ddd, $J_{2'a,3'}$ = 3.7 Hz, $J_{1',2'a}$ = 6.6 Hz, $J_{2'a,2'b}$ = 13.6 Hz, 1 H, H-2'a), 2.25 (app. dt, J = 7.0 Hz, J = 13.6 Hz, 1 H, H-2'b), 3.55 (d, $J_{4',5'}$ = 5.1 Hz, 2 H, H-5'a, H-5'b), 3.84 (app. dt, J = 3.7 Hz, J = 5.1 Hz, 1 H, H-4'), 4.18-4.21 (m, 1 H, H-3'), 5.40 (d, $J_{3',OH}$ = 4.4 Hz, 1 H, OH), 6.20 (dd, $J_{1',2'a}$ = 6.6 Hz, $J_{1',2'b}$ = 7.0 Hz, 1 H, H-1'), 7.49 (d, $J_{CH3T,6}$ = 1.2 Hz, 1 H, H-6), 11.32 (s, 1 H, NH) ppm.
^{13}C {^1H} NMR (151 MHz, DMSO-d_6): δ = 12.1 (CH_3^T), 38.1 (C-2'), 51.7 (C-5'), 70.7 (C-3'), 83.9 (C-1'), 84.6 (C-4'), 109.8 (C-5), 136.1 (C-6), 150.5 (C-2), 163.7 (C-4) ppm.
HRMS ($APCI^-$): m/z calc. 266.0894 $[M - H]^-$, found: 266.0904
The spectroscopic data are in agreement with those reported in the literature.[5]

5′-Amino-5′-deoxythymidine (13)

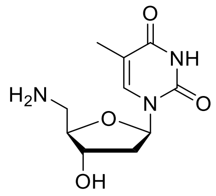

13

Azide **18** (1.68 g, 6.3 mmol) was dissolved in THF (45 mL) under argon. After PPh_3 (3.50 g, 13.4 mmol) was added, the reaction mixture was stirred at r.t. for 10 min before H_2O (15 mL) was added. After stirring at r.t. for 4 h, TLC analysis showed the complete consumption of the starting material (EtOAc-MeOH, 9:1, $R_f = 0.7$) and the formation of the product (H_2O-*i*-PrOH-EtOAc, 1:2:2, $R_f = 0.2$). THF was removed *in vacuo* and the remaining aqueous mixture was extracted with CH_2Cl_2 (5 x 30 mL). The organic layer was washed with H_2O (50 mL) and the combined aqueous layers were concentrated to afford the desired product **13** as a white solid (1.47 g, 97%).

ν_{max}/cm^{-1} (neat) 3349 (N-H, OH), 3315 (N-H), 2930 (C-H), 1658 (C=O), 1605 (C=C).
^1H NMR (400 MHz, DMSO-d_6): δ = 1.78 (d, $J_{CH3T,6}$ = 1.0 Hz, 3 H, CH_3^T), 2.03 (ddd, $J_{2'a,3'}$ = 3.5 Hz, $J_{1',2'a}$ = 6.3 Hz, $J_{2'a,2'b}$ = 13.4 Hz, 1 H, H-2'a), 2.13 (ddd, $J_{2'b,3'}$ = 6.4 Hz, $J_{1',2'b}$ = 7.5 Hz, $J_{2'a,2'b}$ = 13.4 Hz, 1 H,

H-2'b), 2.68 - 2.76 (m, 2 H, H-5'a, H-5'b), 3.64 (app. dt, J = 3.4 Hz, J = 5.2 Hz, 1 H, H-4'), 4.19 (app. dt, J = 3.5 Hz, J = 6.4 Hz, 1 H, H-3'), 5.16 (bs, 3 H, NH_2, OH), 6.13 (dd, $J_{1',2'a}$ = 6.3 Hz, $J_{1',2'b}$ = 7.5 Hz, 1 H, H-1'), 7.64 (d, $J_{CH3T,6}$ = 1.0 Hz, 1 H, H-6) ppm.
^{13}C {^{1}H} NMR (100 MHz, DMSO-d$_6$): δ = 12.6 (CH_3^T), 39.3 (C-2'), 44.1 (C-5'), 71.2 (C-3'), 83.8 (C-1'), 88.4 (C-4'), 110.0 (C-5), 136.7 (C-6), 151.0 (C-2), 164.2 (C-4) ppm.
HRMS (ESI$^+$): m/z calc. 242.1135 [M - H]$^+$, found: 242.1137
The spectroscopic data are in agreement with those reported in the literature.[4]

5'-Amino-5'-*N*-(2-ethoxy-3,4-dioxocyclobuten-1-yl)-5'-deoxythymidine (14)

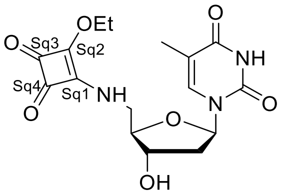

14

Amine **13** (100 mg, 0.41 mmol) was dissolved in DMF (4.0 mL). DIPEA (36 µl, 0.21 mmol) and diethyl squarate (61 µl, 0.41 mmol) were added and the reaction mixture was stirred at r.t. for 24 h. After this time, TLC analysis showed the consumption of the starting material (H_2O-*i*-PrOH-EtOAc, 1:2:2, R_f = 0.2) and the formation of the product (CH_2Cl_2-MeOH, 9:1, R_f = 0.3). The reaction mixture was concentrated and purified by flash column chromatography (EtOAc-MeOH, 9:1) to afford the desired product **14** as a yellow solid (93 mg, 61%).

*Note: Compound **14** exhibits rotamers in NMR spectroscopy.*

ν_{max}/cm^{-1} (neat) 3450 (O-H), 3313 (N-H), 3191 (N-H), 3053 (N-H), 2927 (C-H), 1804 (C=O), 1685 (C=O), 1580 (C=C).
^{1}H NMR (400 MHz, DMSO-d$_6$): δ = 1.32 (t, $J_{CH2Et,CH3Et}$ = 7.0 Hz, 1.5 H, CH_3^{Et}), 1.37 (t, $J_{CH2Et,CH3Et}$ = 7.0 Hz, 1.5 H, CH_3^{Et}), 1.76 (s, 1.5 H, CH_3^T), 1.78 (s, 1.5 H, CH_3^T) 2.04-2.10 (m, 1 H, H-2'a), 2.12-2.22 (m, 1 H, H-2'b), 3.44-3.48 (m, 0.5 H, H-5'a), 3.55-3.58 (m, 0.5 H, H-5'a), 3.70-3.73 (m 1 H, H-5'b), 3.76-3.82 (m, 1 H, H-4'), 4.16-4.20 (m, 0.5 H, H-3'), 4.22-4.26 (m, 0.5 H, H-3'), 4.56-4.68 (m, 2 H, CH_2^{Et}), 5.37 (d, $J_{3',OH}$ = 4.6 Hz, 0.5 H, OH), 5.38 (d, $J_{3',OH}$ = 4.6 Hz, 0.5 H, OH), 6.13-6.16 (m, 1 H, H-1'), 7.33 (s, 0.5 H, H-6), 7.36 (s, 0.5 H, H-6), 8.75 (t, $J_{NHSq,5'a}$ = 5.6 Hz, $J_{NHSq,5'b}$ = 5.6 Hz, 0.5 H, NH), 8.95 (t, $J_{NHSq,5'a}$ = 5.6 Hz, $J_{NHSq,5'b}$ = 5.6 Hz, 0.5 H, NH^{Sq}), 11.31 (bs, 1 H, NH^T) ppm.
^{13}C {^{1}H} NMR (100 MHz, DMSO-d$_6$): δ = 12.0 (CH_3^T), 12.1 (CH_3^T), 15.6 (CH_3^{Et}), 38.1 (C-2'), 38.4 (C-2'), 45.7 (C-5'), 68.86 (CH_2^{Et}), 68.90 (CH_2^{Et}), 70.5 (C-3'), 70.6 (C-3'), 83.68 (C-1'), 83.75 (C-1'), 84.7 (C-4'), 84.9 (C-4'), 109.7 (C-5), 109.8 (C-5), 135.8 (C-6), 136.0 (C-6), 150.4 (C-2), 163.7 (C-4), 172.5 (C-Sq1), 173.2 (C-Sq1), 176.8 (C-Sq2), 177.3 (C-Sq2), 182.1 (C-Sq3), 182.4 (C-Sq3), 189.1 (C-Sq4), 189.4 (C-Sq4), ppm.
HRMS (APCI$^+$): m/z calc. 366.1296 [M + H]$^+$, found: 366.1290

5′-Amino-5′-*N*-(2-ethoxy-3,4-dioxocyclobuten-1-yl)-5′-deoxythymidine-3′-2-cyanoethyl diisopropylphosphoramidite (15)

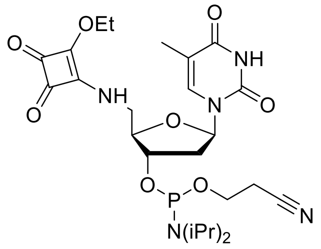

15

Alcohol **14** (90 mg, 246 µmol) was coevaporated with dry CH_2Cl_2 (3 x 2.0 mL), dried under high vacuum and suspended in dry THF (1.5 mL) under argon. Molecular sieves (3 Å) were added followed by the addition of DIPEA (108 µL, 620 µmol). The flask was purged with argon and 2-Cyanoethyl *N,N*-diisopropylchlorophosphoramidite (70 µL, 314 µmol) in dry THF (0.4 mL) was added dropwise and the reaction mixture was stirred for 2 h. After this time, TLC analysis (EtOAc-pyridine, 99.5:0.5) showed the consumption of the starting material (R_f = 0.1) and the formation of the product (R_f = 0.6). The solution was concentrated and dissolved in dry $CHCl_3$ (20 mL). The suspension was washed with sat. aq. KCl (10 mL), dried over Na_2SO_4 and concentrated. Purification by flash column chromatography (EtOAc-pyridine 99.5:0.5) afforded the desired product **15** as an oil (98 mg, 70%). The formation of the phosphoramidite was confirmed by ^{31}P NMR and the phosphoramidite was used in solid phase synthesis without further characterisation.

^{31}P NMR (162 MHz, DMSO-d_6): 147.4, 147.56, 147.61, 147.64 ppm.
HRMS (ESI$^+$): *m/z* calc. 588.2194 [M + Na]$^+$, found: 588.2210

2. NMR spectra

N-acetyl-5'-*O*-amino-3'-*O*-(*tert*-butyldimethylsilyl)thymidine (17)

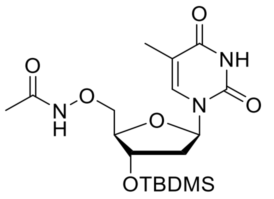

17

^{1}H NMR – $CDCl_3$

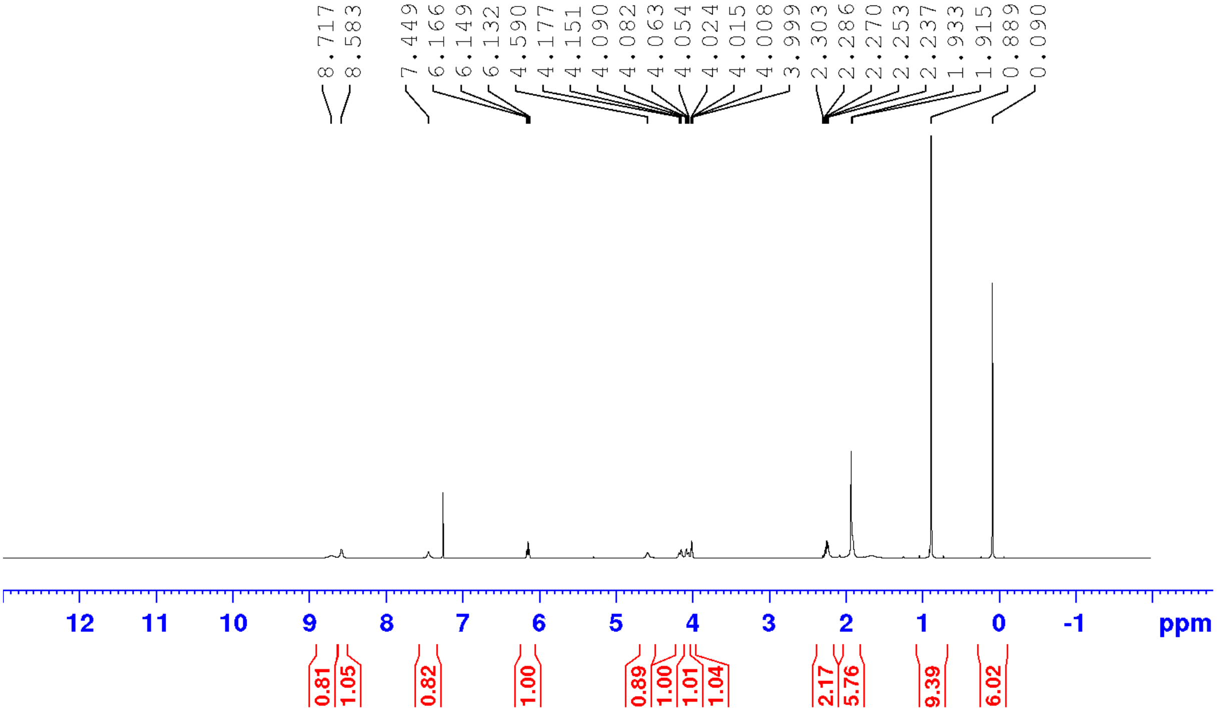

^{13}C NMR – $CDCl_3$

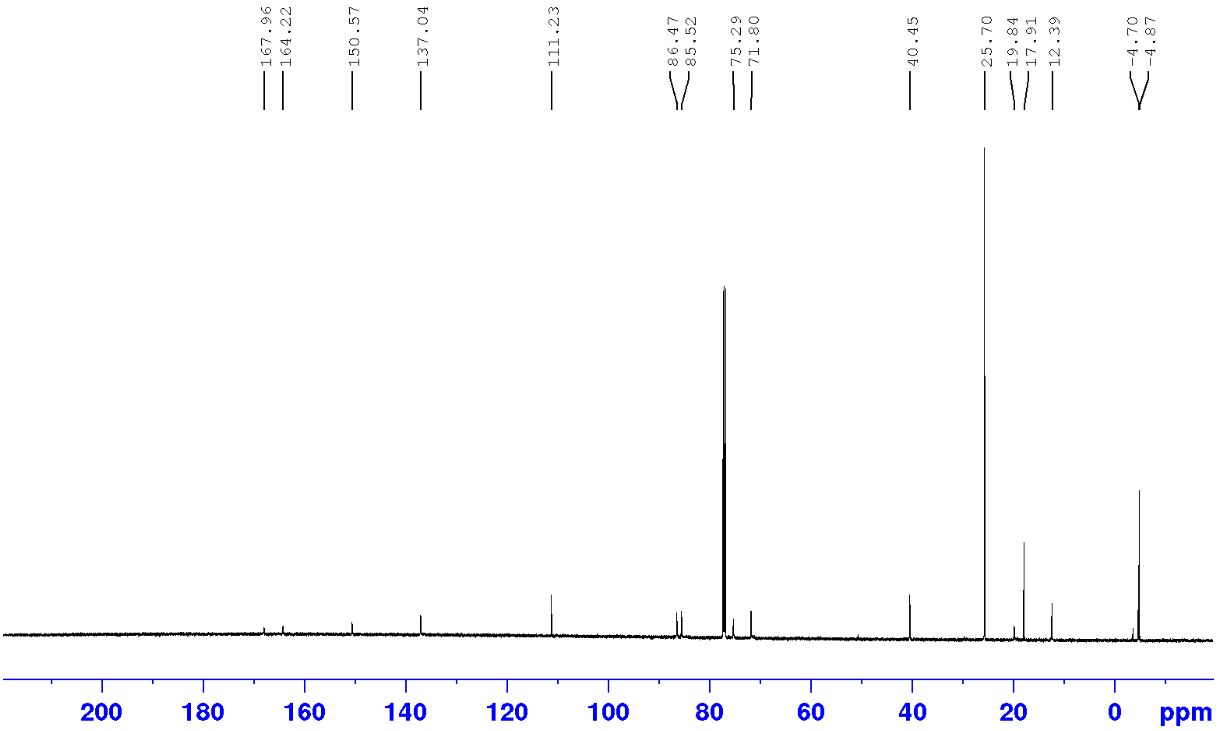

_N_-acetyl-5'-_O_-aminothymidine (10)

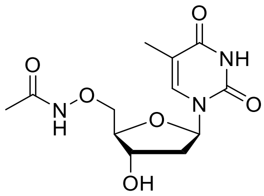

10

^{1}H NMR – DMSO-d$_6$

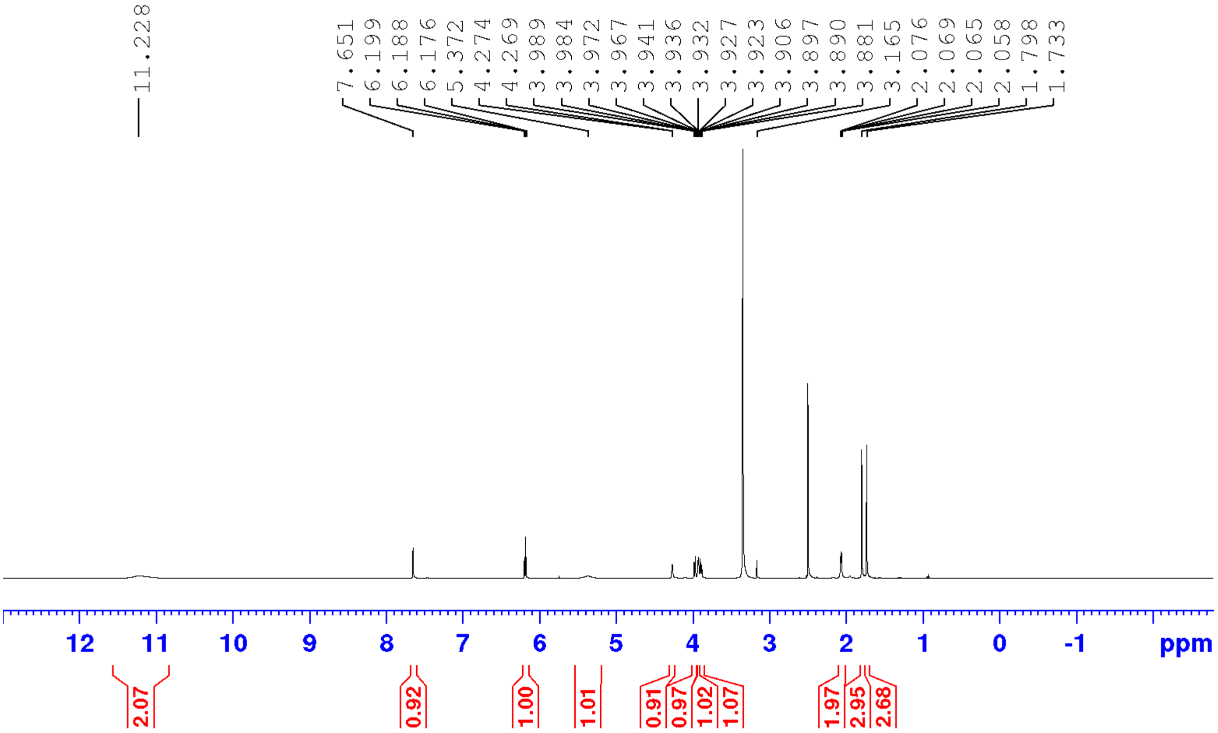

^{13}C NMR – DMSO-d$_6$

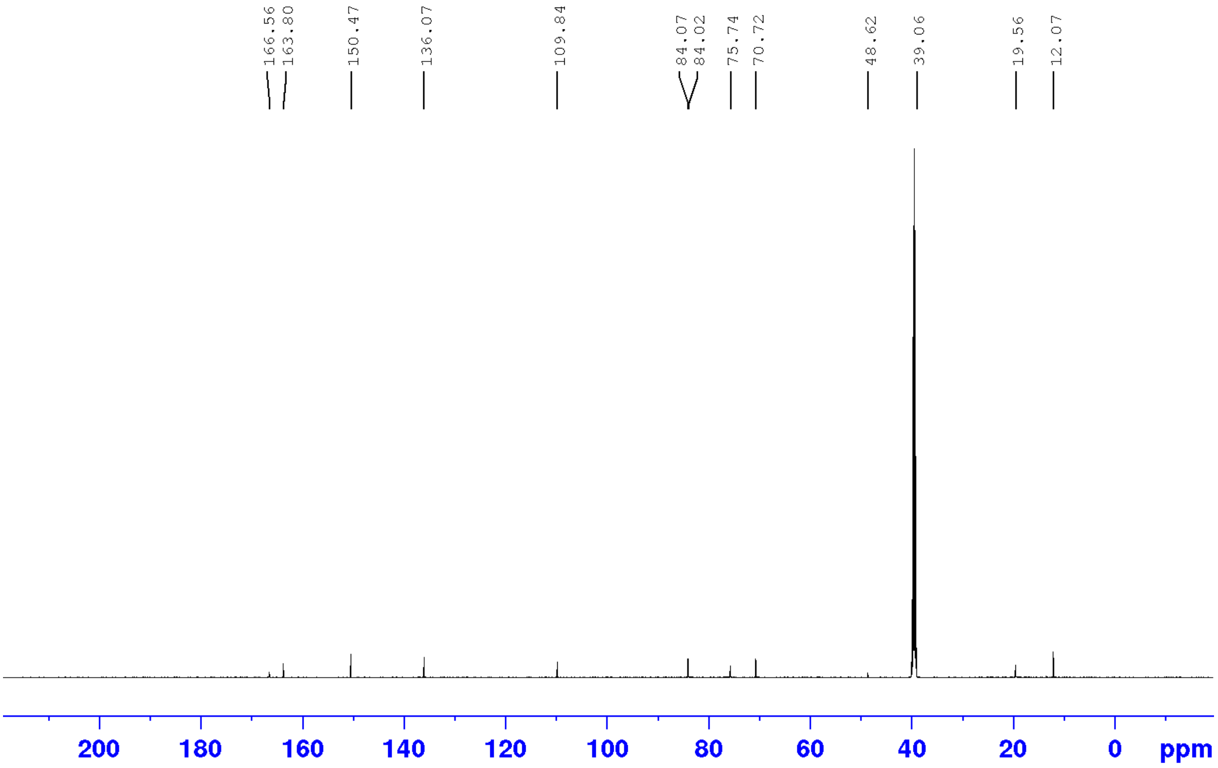

5’-Deoxy-5’-iodothymidine (12)

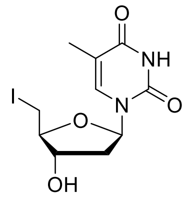

12

^{1}H NMR – DMSO-d6

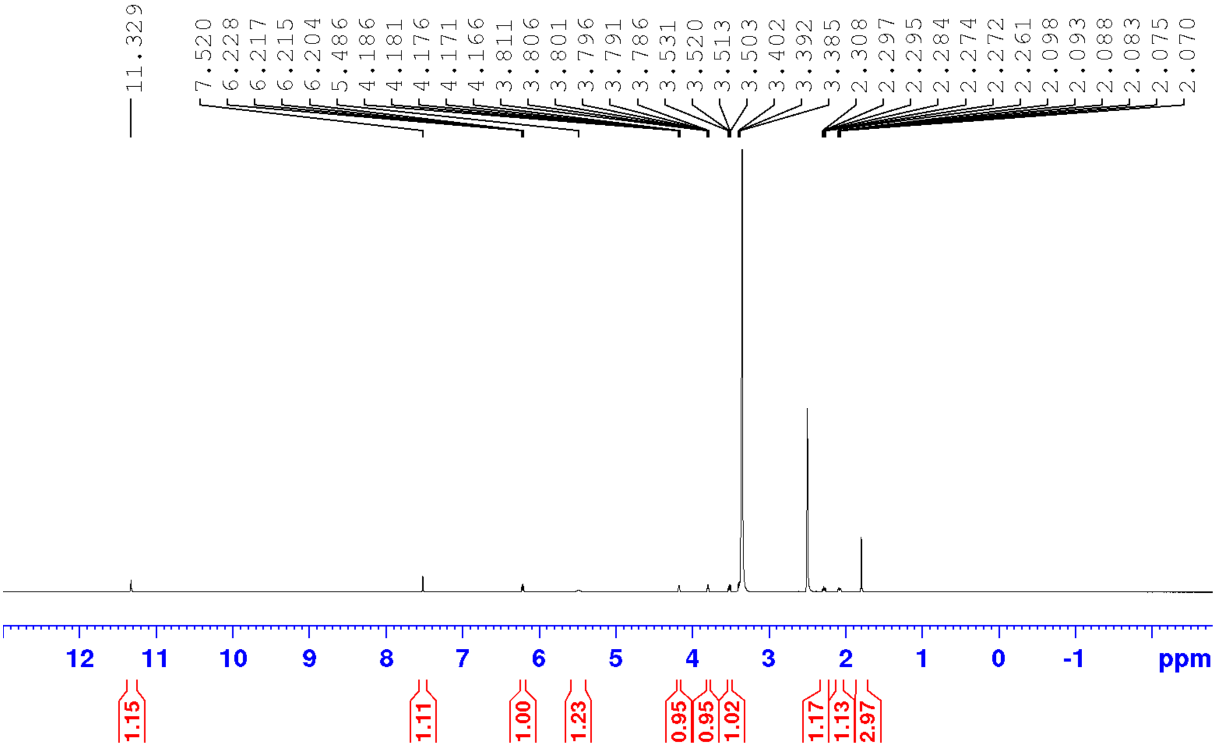

^{13}C NMR – DMSO-d6

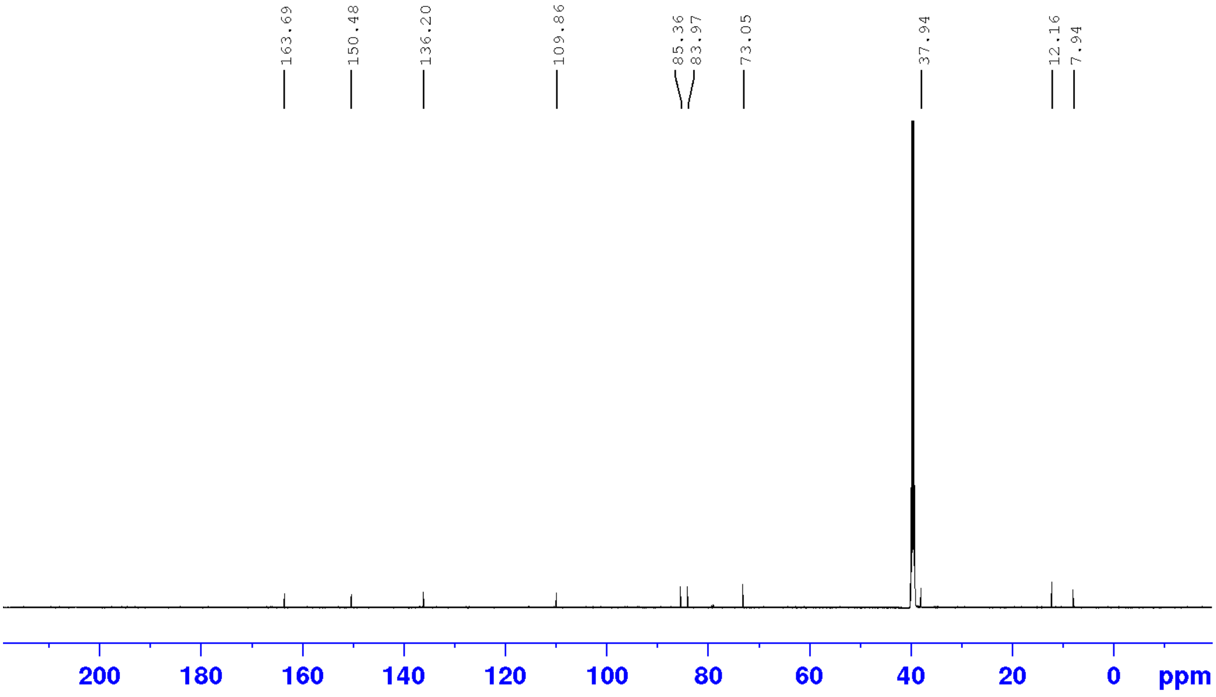

5’-Azido-5’-deoxythymidine (18)

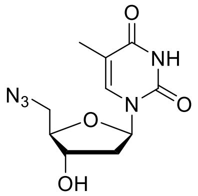

18

^{1}H NMR - DMSO-d_6

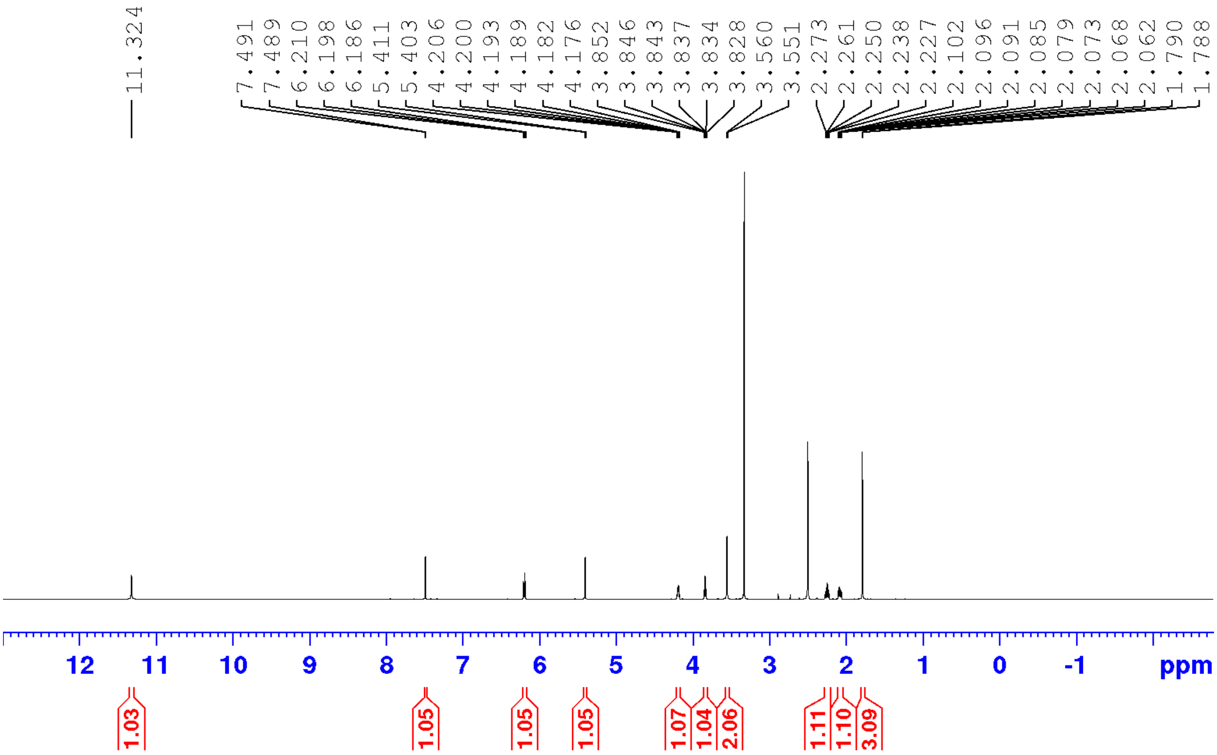

^{13}C NMR – DMSO-d_6

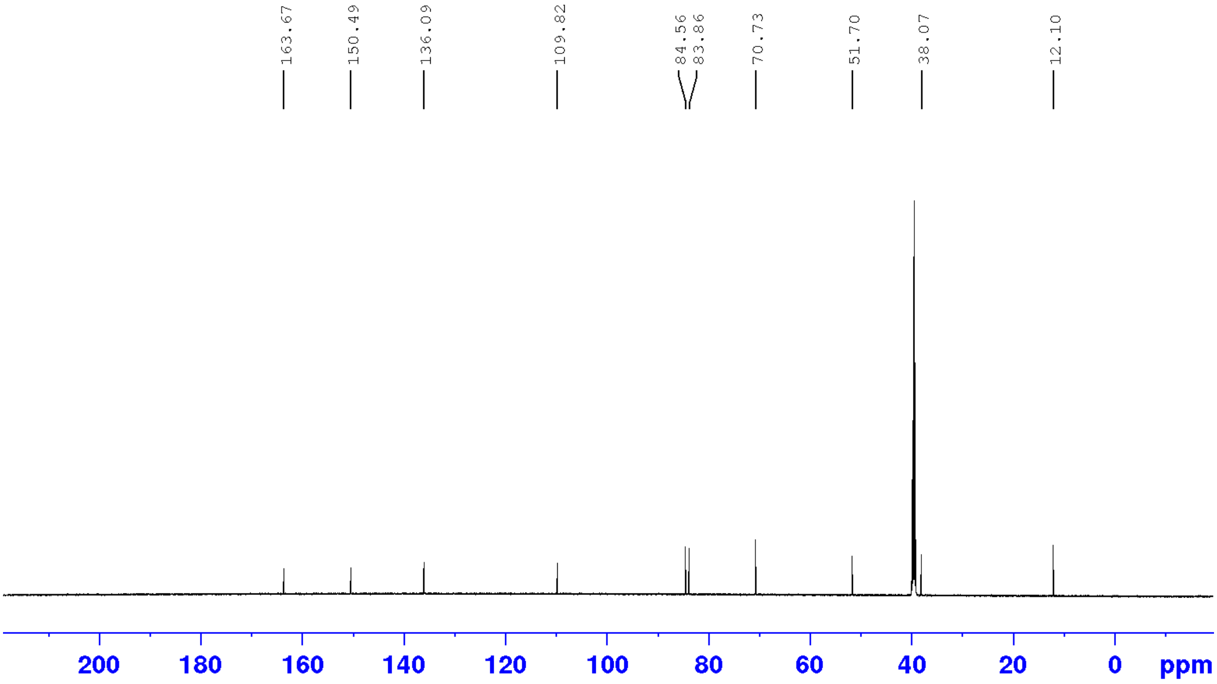

5′-Amino-5′-deoxythymidine (13)

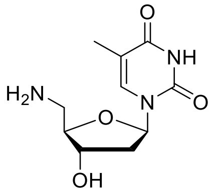

13

^{1}H NMR – DMSO-d_6

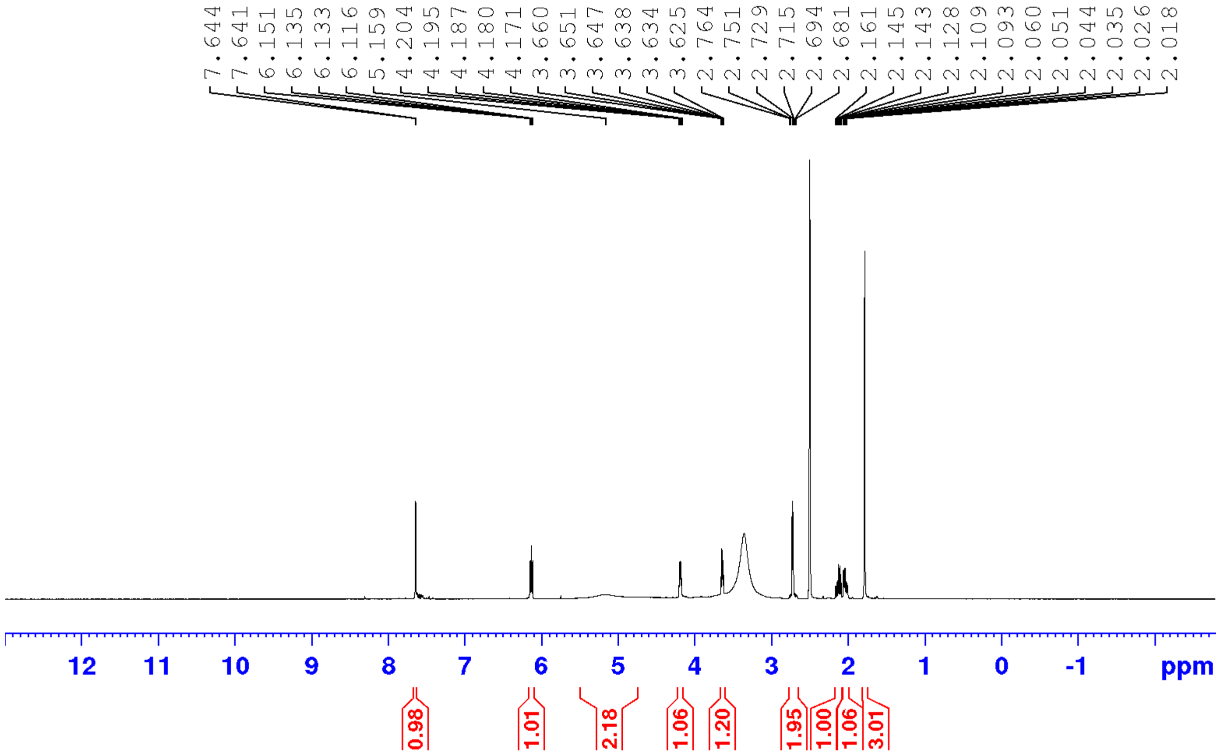

^{13}C NMR – DMSO-d_6

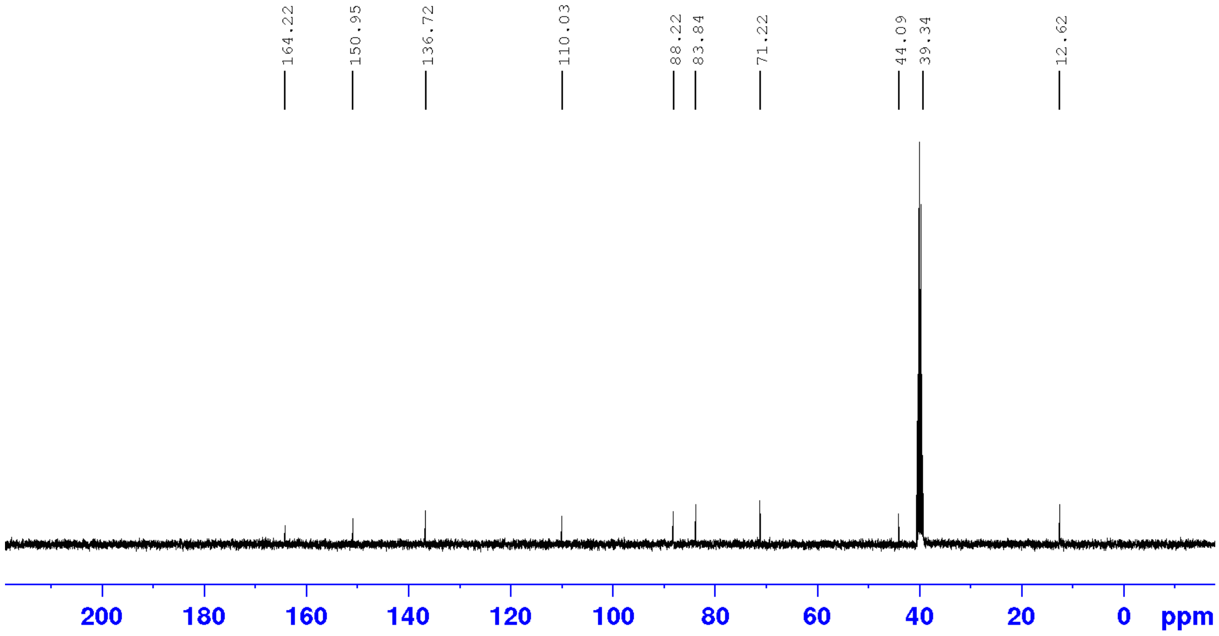

5’-Amino-5’-*N*-(2-ethoxy-3,4-dioxocyclobuten-1-yl)-5’-deoxythymidine (14)

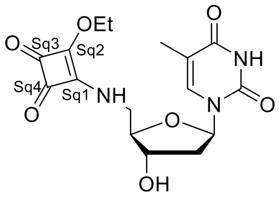

14

^{1}H NMR – DMSO-d$_6$

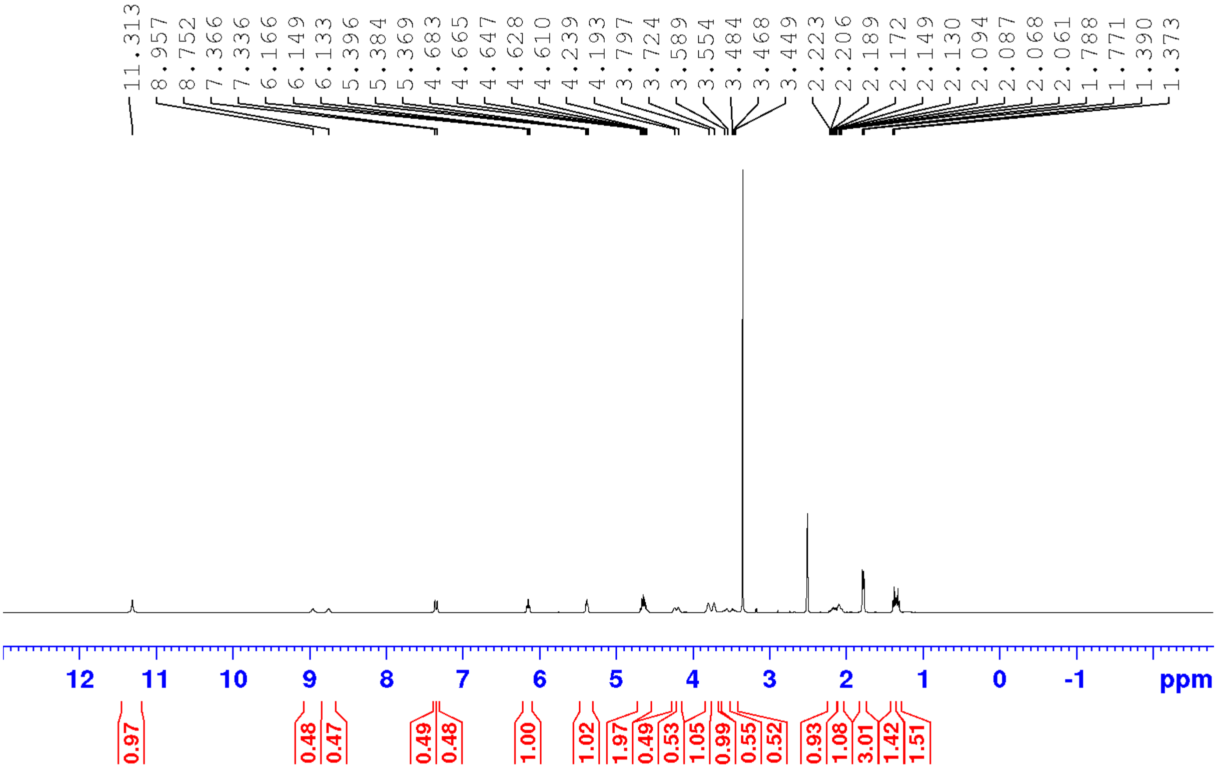

^{13}C NMR – DMSO-d$_6$

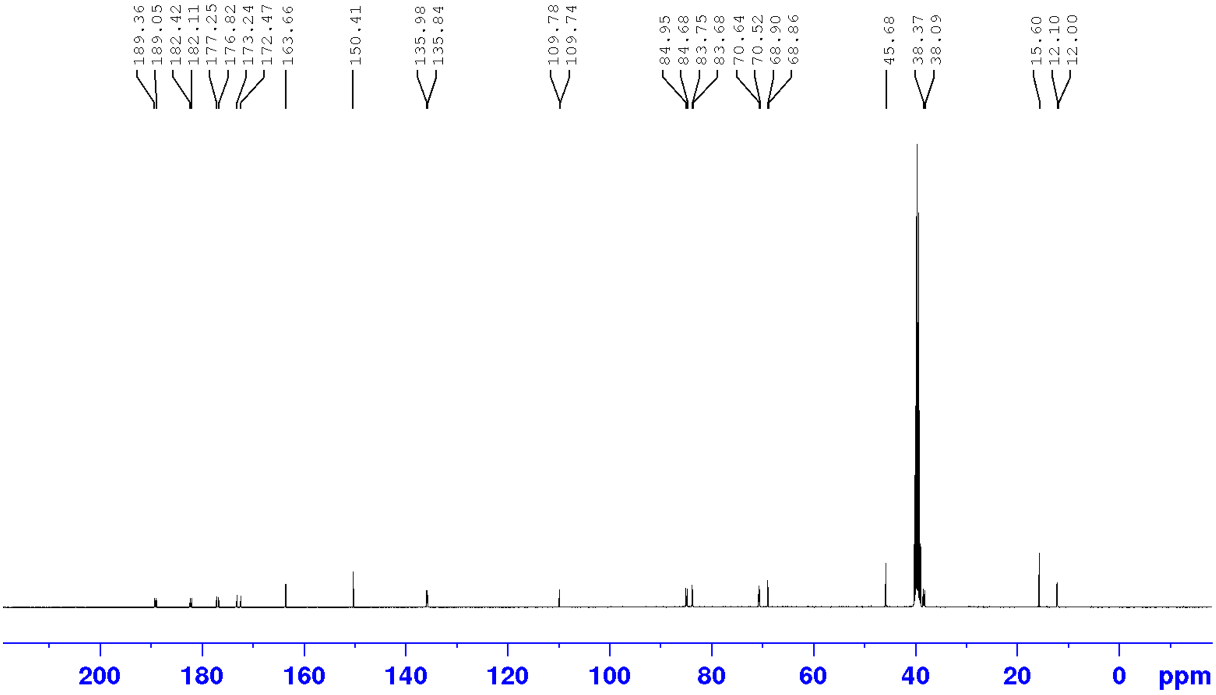

5'-Amino-5'-*N*-(2-ethoxy-3,4-dioxocyclobuten-1-yl)-5'-deoxythymidine-3'-2-cyanoethyl diisopropylphosphoramidite (15)

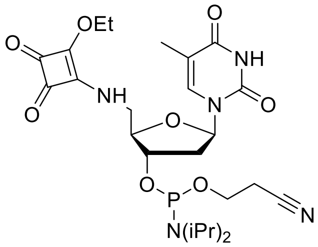

15

^{31}P NMR – DMSO-d$_6$

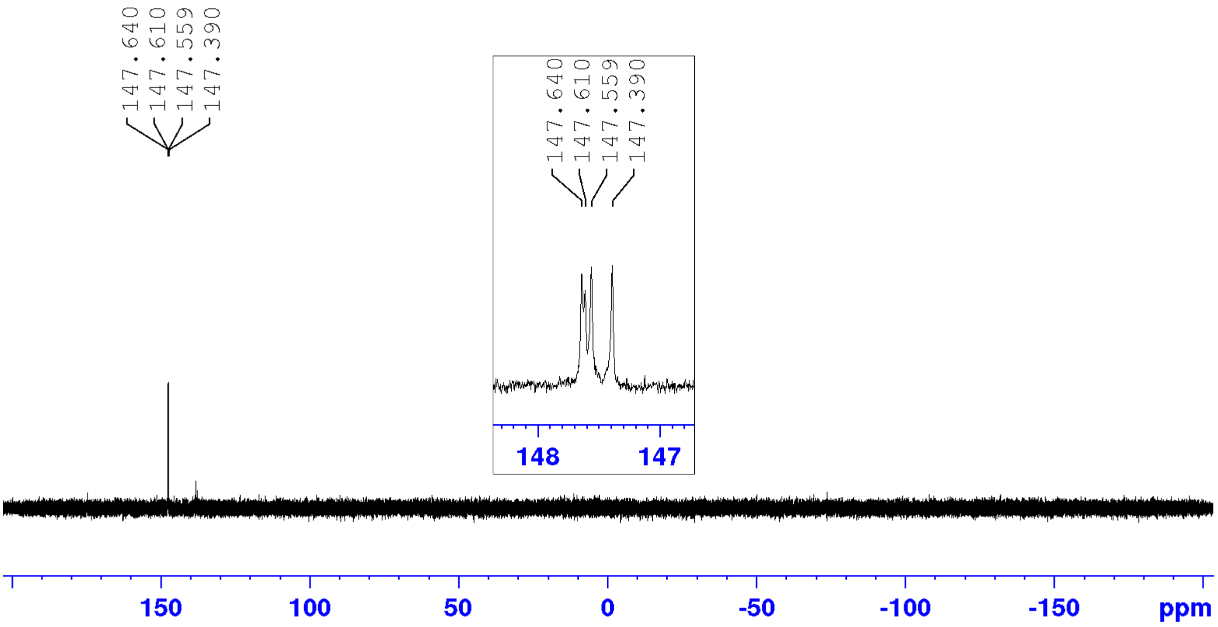

3. Oligonucleotide synthesis

Oligonucleotides of the sequence 5′-XTAG CAG TCA GTC AGT CAT CGY-3′, where X = modified nucleoside or thymidine and Y = Cy3 or OH, were synthesised using solid-phase oligonucleotide synthesis. Standard DNA phosphoramidites, solid supports and reagents were purchased from Link Technologies and Applied Biosystems. Automated solid phase synthesis of oligonucleotides was performed on an Applied Biosystems 394 synthesiser. Synthesis was performed on 1.0 µmol scale involving cycles of acid-catalysed detritylation, coupling, capping, and iodine oxidation. All 2-cyanoethyl phosphoramidite monomers were dissolved in dry MeCN to a concentration of 0.1 M immediately before use. Standard DNA phosphoramidites were coupled for 60 s while extended coupling time of 10 min was used for modified phosphoramidites. Coupling efficiencies and overall synthesis yields, excluding coupling of the modified nucleotides, were determined by the inbuilt automated trityl cation conductivity monitoring facility and were ≥ 98.0% in all cases. The oligonucleotides, with the exception of 5′-deoxyamino oligonucleotides, were then cleaved from the solid support and protecting groups from the nucleobase and backbone were removed by heating in conc. aq. NH_4OH in a sealed tube for 5 h at 55 °C. 5′-Deoxyamino oligonucleotides were deprotected by washing with Et_2NH in MeCN (20%) followed by 0.4 M NaOH in MeOH-H_2O 4:1 for 10 min at r.t. and 2.5 h at 55 °C and desalted using NAP-5 columns. The oligonucleotides were purified by reversed-phase HPLC on a Gilson system using a Luna 10 µ C8 100Å pore Phenomenex column (10 x 250 mm) with a gradient of MeCN in triethylammonium bicarbonate (0% to 50% over 12.5 min, flow rate 4 mL/min), (buffer A: 0.1 M triethylammonium bicarbonate, pH 7.6, buffer B: 0.1 M triethylammonium bicarbonate, pH 7.6, with 50% MeCN). Elution was monitored by UV absorption at 290 nm or 298 nm. Electrospray mass spectrometry of oligonucleotides was performed in H_2O using a Waters ESI HRMS. Data were processed using MaxEnt. Oligonucleotide concentrations were determined by measuring absorbance in H_2O at 260 nm.

Synthesis of squaramide-containing oligonucleotides. 5′-amino oligonucleotide **16a-b** was dissolved in 1.0 M sodium borate buffer (1.0 mL, pH 8.5). Aqueous dimethyl squarate solution (2 mL, 300 eq.) was added and the solution was left at r.t. for 3 h. After this time, unreacted dimethyl squarate and salts were removed by NAP-10 column (GE Healthcare) according to manufacturer instructions to give 5′-squaryl monoamide oligonucleotides **2a-b**.
A solution of 5′-squaryl monoamide oligonucleotide **2a-b** in H_2O (20 µL) was dissolved in a solution of Et_2NH in H_2O (125 mM, 80 µL) or $MeNH_2$ in H_2O (125 mM, 80 µL) and the solution was left at r.t. for 4 h. After this time, unreacted Et_2NH or $MeNH_2$ was removed by NAP-5 column according to manufacturer instructions to give 5′-squaryl diamide oligonucleotides **3a-b** or **4a-b**.

Phosphorylation of oligonucleotide. 5′-OH oligonucleotide **6a** (0.3 nmol), ATP (50 nmol) and T4 polynucleotide kinase (10 units, New England Biolabs) were incubated in kinase reaction buffer (50 µL; 10 mM $MgCl_2$, 70 mM Tris HCl, 5 mM DTT, pH 7.6) at 37 °C for 30 min according to manufacturer instructions. The enzyme was inactivated by heating to 65 °C for 20 min to give the 5′-phosphorylated oligonucleotide **5a**.

Oligonucleotide	5'-Modification X	3'-Modificaction Y	Mass (calc.)
**1a**	5'-oxyamide thymidine	Cy3	6984 (6985)
**1b**	5'-oxyamide thymidine	OH	6477 (6478)
**2a**	5'-squaryl monoamide thymidine	Cy3	7036 (7037)
**2b**	5'-squaryl monoamide thymidine	OH	6530 (6530)
**3a**	5'-squaryl diamide thymidine (Et_2NR)	Cy3	7077 (7078)
**3b**	5'-squaryl diamide thymidine (Et_2NR)	OH	6571 (6571)
**4a**	5'-squaryl diamide thymidine (MeNHR)	Cy3	7036 (7036)
**4b**	5'-squaryl diamide thymidine (MeNHR)	OH	6528 (6529)
**6a**	thymidine	Cy3	6927 (6928)
**6b**	thymidine	OH	6420 (6421)

Table S1. Mass spectroscopy data of modified oligonucleotides **1-4** and **6** of the sequence 5'-XTAG CAG TCA GTC AGT CAT CGY-3'.

4. Mass spectra of oligonucleotides

5'-*N*-oxyamide-3'-Cy3 oligonucleotide (1a)

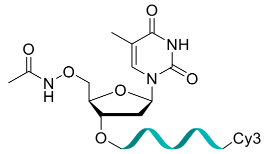

5'-*N*-oxyamide-3'-OH oligonucleotide (1b)

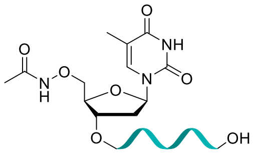

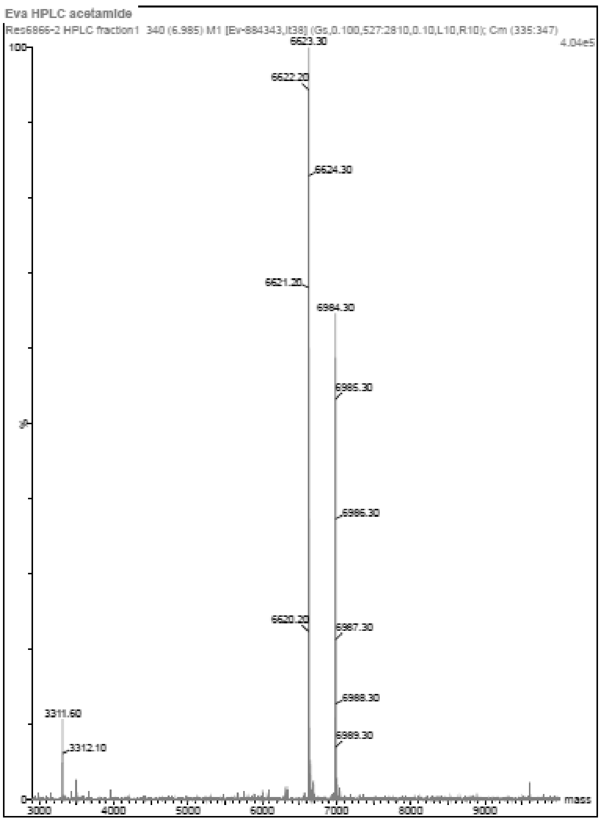

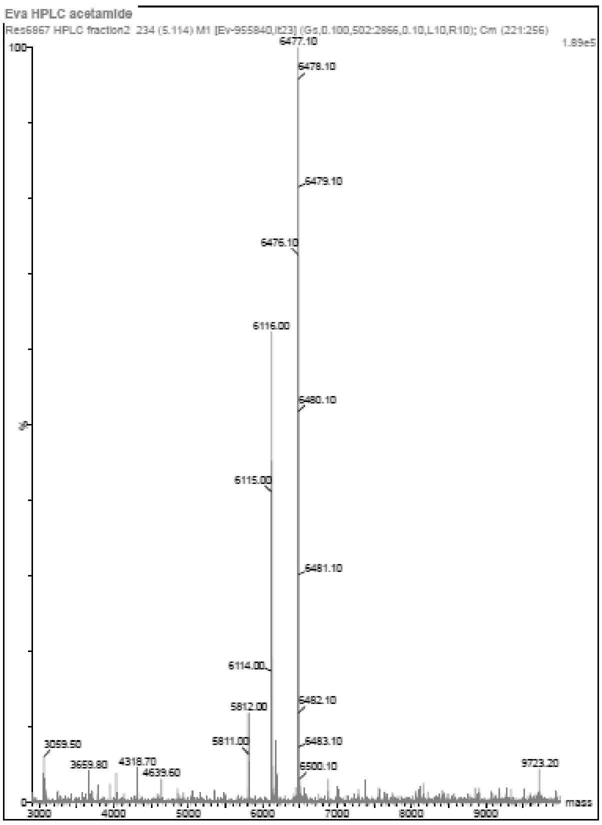

5′-Squaryl monoamide-3′-Cy3 oligonucleotide (2a)

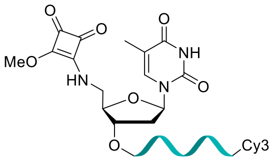

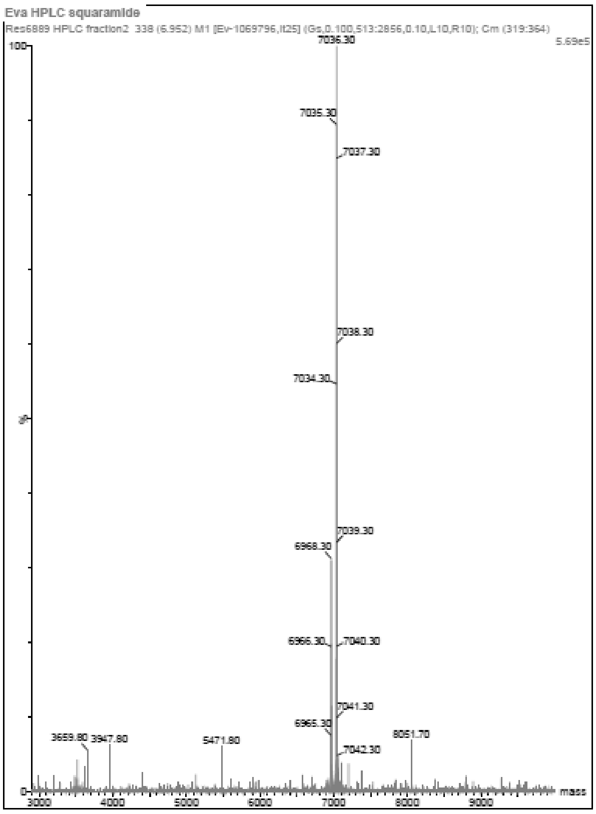

5′-Squaryl monoamide-3′-OH oligonucleotide (2b)

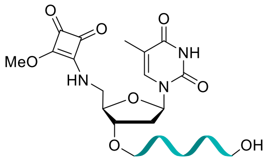

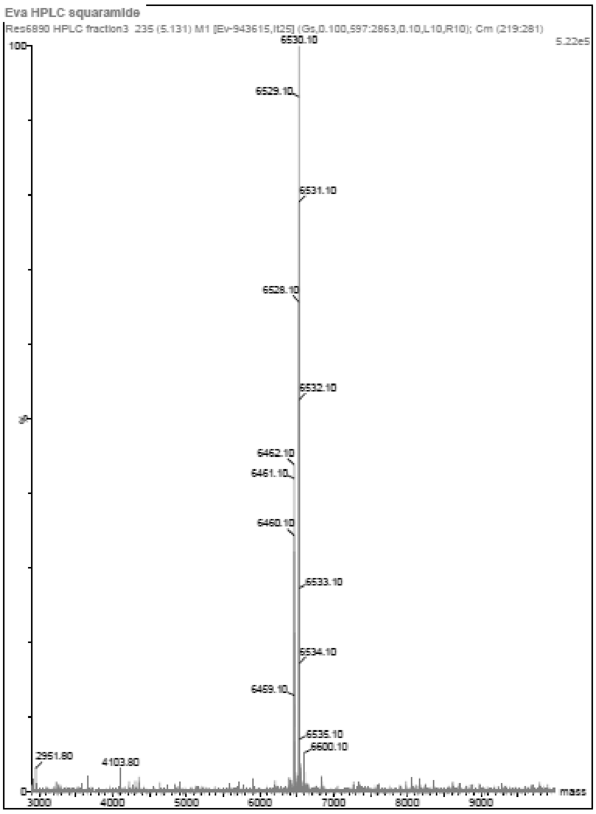

5’-Squaryl diamide-3’-Cy3 oligonucleotide (3a)

5’-Squaryl diamide-3’-OH oligonucleotide (3b)

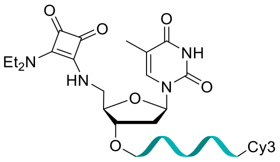

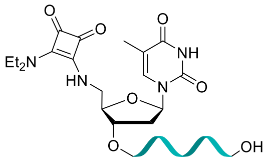

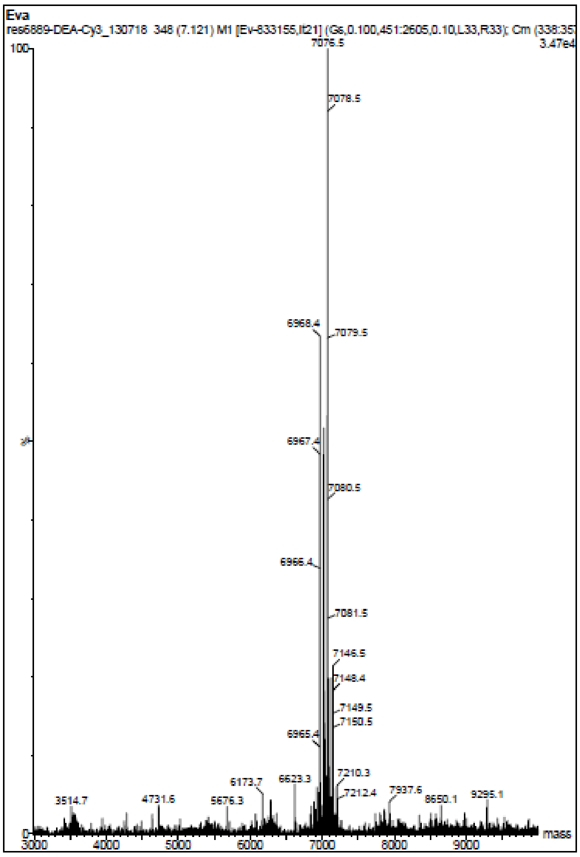

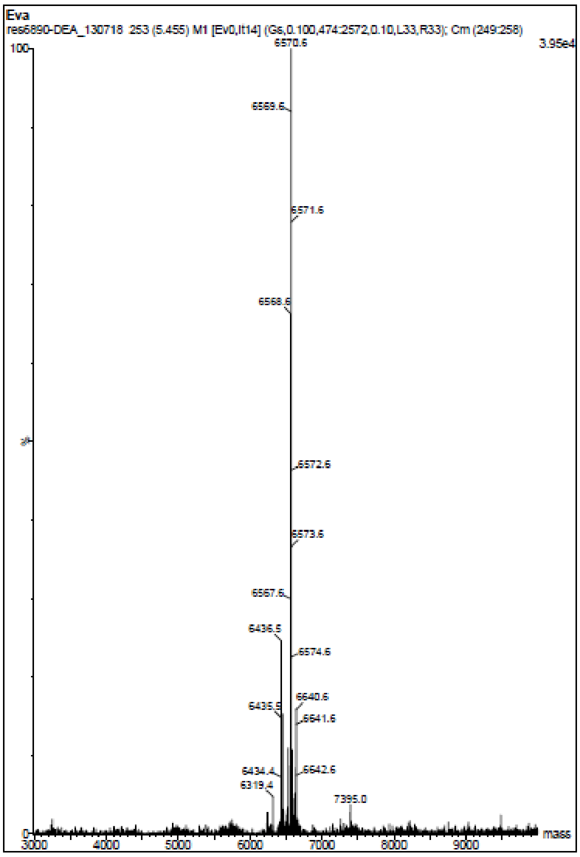

5′-Squaryl diamide-3′-Cy3 oligonucleotide (4a)

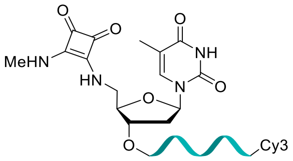

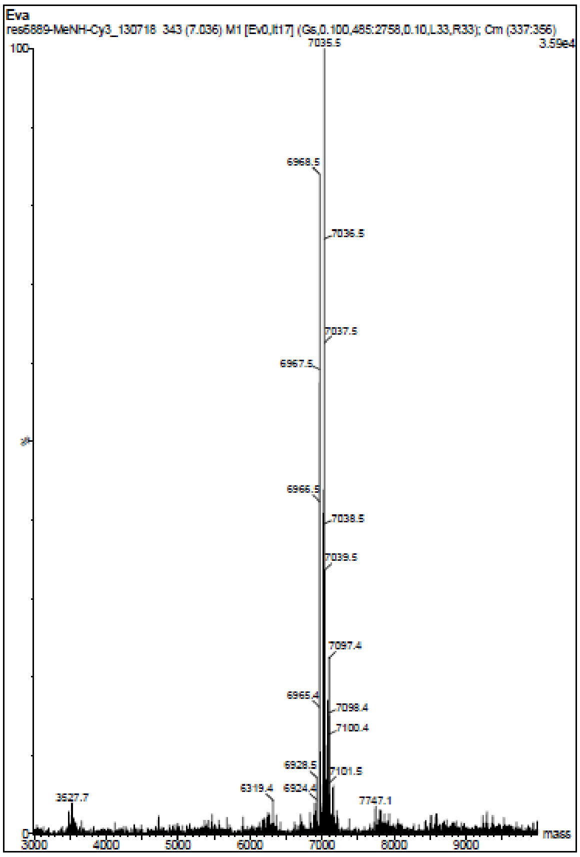

5′-Squaryl diamide-3′-OH oligonucleotide (4b)

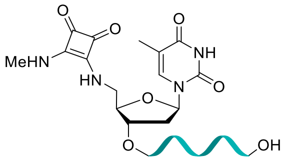

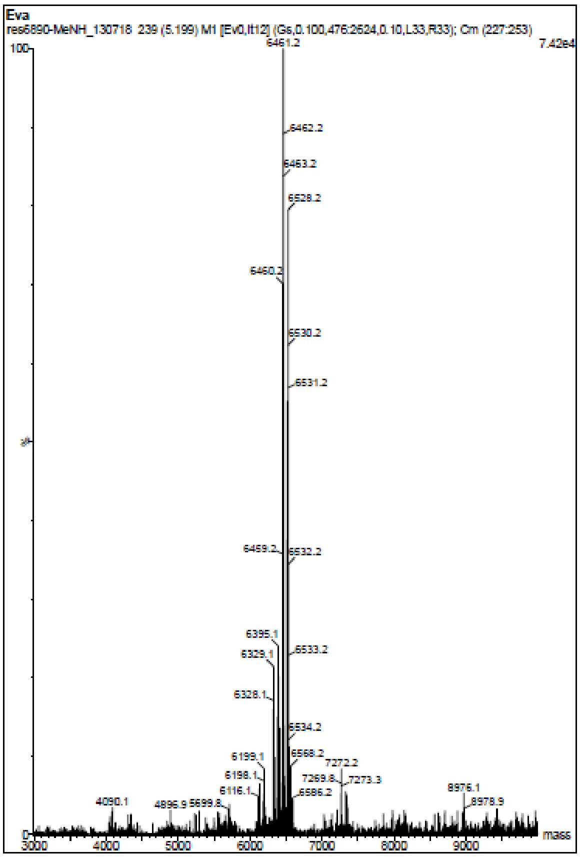

5’-OH-3’-Cy3 oligonucleotide (6a)

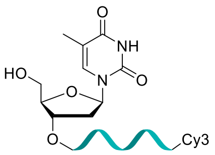

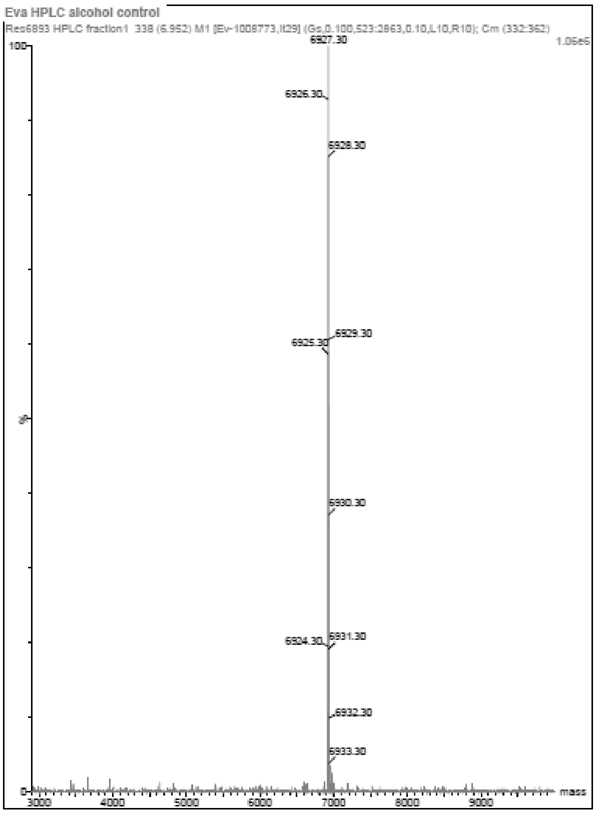

5’-OH-3’-OH oligonucleotide (6b)

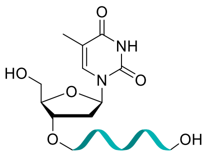

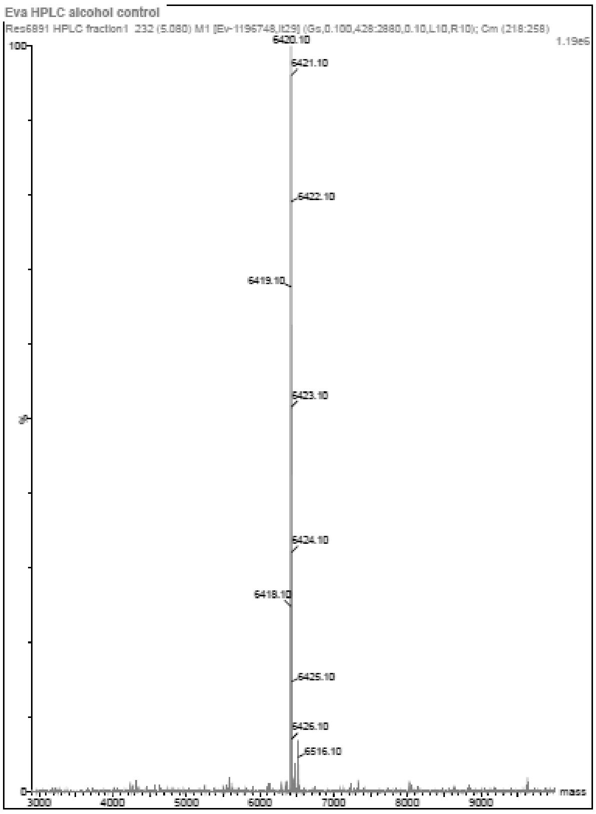

5. Biological evaluation – procedures and supplementary figures

Hydrolysis assay with SNM1A. ΔN-SNM1A (698-1040) was purified as described in Allerston et al.[S6] SNM1A was stored as a 1.0 µM solution in reaction buffer (20 mM HEPES-KOH, pH 7.5, 50 mM KCl, 10 mM $MgCl_2$, 0.05% Triton-X, 0.1 mg mL^{-1} BSA, 5% glycerol, 0.5 mM DTT). Oligonucleotides **1a-5a** (0.8 pmol or as specified) were mixed with SNM1A (25 fmol or as specified) in reaction buffer (10 µL) on ice and incubated at 37 °C for 60 min or as specified. The reaction was stopped by the addition of stop solution (2 µL, 95% formamide, 10 mM EDTA) followed by heating to 95 °C for 3 min. Oligonucleotides were separated on a 15% acrylamide 6.5 M urea gel (2.9 g urea, 2.7 mL 40% acrylamide-bisacrylamide 25:1, 1.4 mL 5X TBE (0.45 M Tris, 0.45 M boric acid, 0.01 M EDTA pH 8.0), 0.6 mL H_2O) in 1X TBE at 150 V for 60 min alongside bromophenol blue and xylene cyanol as markers for 8 nt and 28 nt respectively, and imaged using a Typhoon FLA 9500.

Inhibition assay with SNM1A. Oligonucleotides **1b-4b** or **6b** (0.8 pmol or as specified) were mixed with SNM1A (25 fmol) in reaction buffer (10 µL) on ice and, if specified, incubated at 37 °C for 5 min. Phosphorylated oligonucleotide **5a** (0.8 pmol) was added and the mixture was incubated at 37 °C for a further 60 min or as specified. The reaction was stopped by the addition of stop solution (2 µL, 95% formamide, 10 mM EDTA) followed by heating to 95 °C for 3 min. Oligonucleotides were separated on a 15% acrylamide 6.5 M urea gel (2.9 g urea, 2.7 mL 40% acrylamide-bisacrylamide 25:1, 1.4 mL 5X TBE (0.45 M Tris, 0.45 M boric acid, 0.01 M EDTA pH 8.0), 0.6 mL H_2O) in 1X TBE at 150 V for 75 min alongside bromophenol blue and xylene cyanol as markers for 8 nt and 28 nt respectively, and imaged using Typhoon FLA 9500.

Inhibition assay with SNM1A for quantification. Oligonucleotides **4b** or **6b** (2.7 pmol) were mixed with SNM1A (25 fmol) in reaction buffer (10 µL) on ice and incubated at 37 °C for 5 min. Phosphorylated oligonucleotide **5a** (0.8 pmol) was added and the mixture was incubated at 37 °C for a further 10 min. The reaction was stopped by the addition of stop solution (2 µL, 95% formamide, 10 mM EDTA) followed by heating to 95 °C for 3 min. Oligonucleotides were separated on a 15% acrylamide 6.5 M urea gel (2.9 g urea, 2.7 mL 40% acrylamide-bisacrylamide 25:1, 1.4 mL 5X TBE (0.45 M Tris, 0.45 M boric acid, 0.01 M EDTA pH 8.0), 0.6 mL H_2O) in 1X TBE at 150 V for 110 min alongside bromophenol blue and xylene cyanol as markers for 8 nt and 28 nt respectively, and imaged using Typhoon FLA 9500. Quantification was carried out using ImageQuant TL.

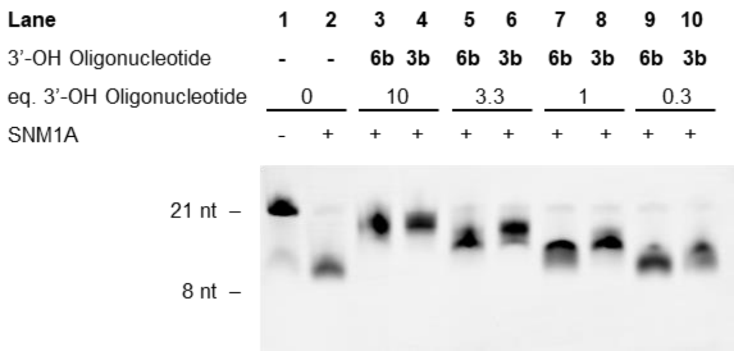

Figure S1: Evaluation of modified oligonucleotides as inhibitors of SNM1A. Digestion of oligonucleotide **5a** by SNM1A (25 fmol, 2.5 nM) in the presence of different amounts of oligonucleotide **3b** or **6b** relative to **5a** (0.8 pmol, 80 nM) after 60 min. nt = nucleotides.

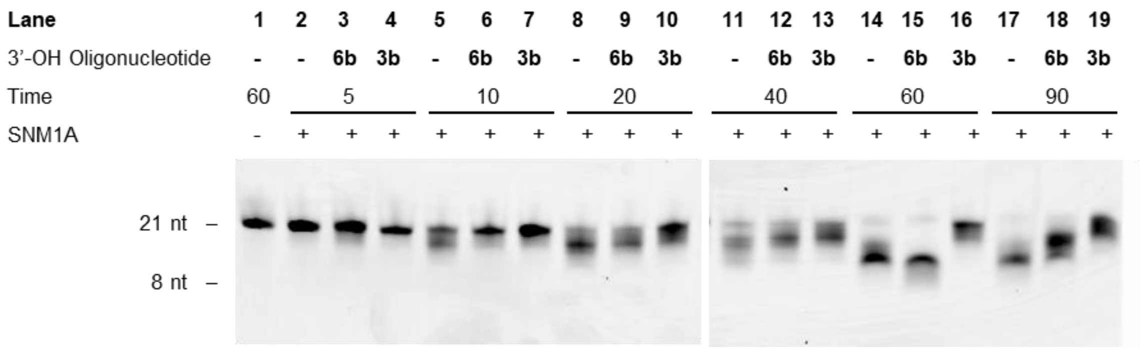

Figure S2: Evaluation of modified oligonucleotides as inhibitors of SNM1A. Digestion of oligonucleotide **5a** by SNM1A (25 fmol, 2.5 nM) in the presence of 3.3 equivalents of oligonucleotide **3b** or **6b** (2.6 pmol, 264 nM) relative to **5a** (0.8 pmol, 80 nM) after varying incubation times. nt = nucleotides.

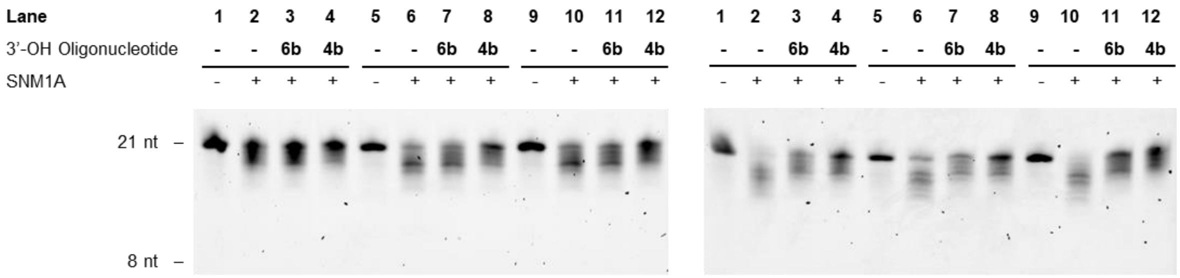

Figure S3: Evaluation of modified oligonucleotide **4b** as an inhibitor of SNM1A. Digestion of oligonucleotide **5a** by SNM1A (25 fmol, 2.5 nM) in the presence of 3.3 equivalents of oligonucleotide **4b** or **6b** (2.7 pmol, 264 nM) relative to **5a** (0.8 pmol, 80 nM) after 10 min with 5 min preincubation. Six experiments shown, performed in triplicate. nt = nucleotides.

References

[1] S. Peyrat, J. Xie, *Synthesis*, **2012**, *44*, 1718-1724.

[2] R. Liboska, J. Snasel, I. Barvik, M. Budesinsky, R. Pohl, Z. Tocik, O. Pav, D. Rejman, P. Novak, I. Rosenberg, *Org. Biomol. Chem.* **2011**, *9*, 8261-8267.

[3] S. K. V. Vernekar, L. Qiu, J. Zacharias, R. J. Geraghty, Z. Wang, *MedChemComm* **2014**, *5*, 603-608.

[4] K. Suthagar, A. J. Fairbanks, *Chem. Commun.* **2017**, *53*, 713–715.

[5] D. Pan, J. Sun, H. Jin, Y. Li, L. Li, Y. Wu, L. Zhang, Z. Yang, *Chem. Commun.* **2015**, *51*, 469-472.

[6] C. K. Allerston, S. Y. Lee, J. A. Newman, C. J. Schofield, P. J. McHugh, O. Gileadi, *Nucleic Acids Res.* **2015**, *43*, 11047-11060.
